# The Conserved Intronic Cleavage and Polyadenylation Site of CstF-77 Gene Imparts Control of 3′ End Processing Activity through Feedback Autoregulation and by U1 snRNP

**DOI:** 10.1371/journal.pgen.1003613

**Published:** 2013-07-11

**Authors:** Wenting Luo, Zhe Ji, Zhenhua Pan, Bei You, Mainul Hoque, Wencheng Li, Samuel I. Gunderson, Bin Tian

**Affiliations:** 1 Department of Biochemistry and Molecular Biology, New Jersey Medical School, University of Medicine and Dentistry of New Jersey, Newark, New Jersey, United States of America; 2 Graduate School of Biomedical Sciences, University of Medicine and Dentistry of New Jersey, Newark, New Jersey, United States of America; 3 Department of Molecular Biology and Biochemistry, Rutgers University, Piscataway, New Jersey, United States of America; Columbia University, United States of America

## Abstract

The human gene encoding the cleavage/polyadenylation (C/P) factor CstF-77 contains 21 exons. However, intron 3 (In3) accounts for nearly half of the gene region, and contains a C/P site (pA) with medium strength, leading to short mRNA isoforms with no apparent protein products. This intron contains a weak 5′ splice site (5′SS), opposite to the general trend for large introns in the human genome. Importantly, the intron size and strengths of 5′SS and pA are all highly conserved across vertebrates, and perturbation of these parameters drastically alters intronic C/P. We found that the usage of In3 pA is responsive to the expression level of CstF-77 as well as several other C/P factors, indicating it attenuates the expression of CstF-77 via a negative feedback mechanism. Significantly, intronic C/P of CstF-77 pre-mRNA correlates with global 3′UTR length across cells and tissues. In addition, inhibition of U1 snRNP also leads to regulation of the usage of In3 pA, suggesting that the C/P activity in the cell can be cross-regulated by splicing, leading to coordination between these two processes. Importantly, perturbation of CstF-77 expression leads to widespread alternative cleavage and polyadenylation (APA) and disturbance of cell proliferation and differentiation. Thus, the conserved intronic pA of the CstF-77 gene may function as a sensor for cellular C/P and splicing activities, controlling the homeostasis of CstF-77 and C/P activity and impacting cell proliferation and differentiation.

## Author Summary

Autoregulation is commonly used in biological systems to control the homeostasis of certain activity, and cross-regulation coordinates multiple processes. We show that vertebrate genes encoding the cleavage/polyadenylation (C/P) factor CstF-77 contain a conserved intronic C/P site (pA) which regulates CstF-77 expression through a negative feedback loop. Since the usage of this intronic pA is also responsive to the expression of other C/P factors, the pA can function as a sensor for the cellular C/P activity. Because the CstF-77 level is important for the usage of a large number of pAs in the genome and is particularly critical for expression of genes involved in cell cycle, this autoregulatory mechanism has far-reaching implications for cell proliferation and differentiation. The human intron harboring the pA is large and has a weak 5′ splice site, both of which are also highly conserved in other vertebrates. Inhibition of U1 snRNP, which recognizes the 5′ splice site of intron, leads to upregulation of the intronic pA isoform of CstF-77 gene, suggesting that the C/P activity in the cell can be cross-regulated by splicing, leading to coordination between these two processes.

## Introduction

Pre-mRNA cleavage/polyadenylation (C/P) is a 3′ end processing mechanism employed by almost all protein-coding genes in eukaryotes [1], [2]. The site for C/P, commonly known as the polyA site or pA, is typically defined by both upstream and downstream cis elements [3], [4]. In metazoans, upstream elements include the polyadenylation signal (PAS), such as AAUAAA, AUUAAA, or close variants, located within ∼40 nucleotides (nt) from the pA; the UGUA element [5], typically located upstream of the PAS; and U-rich elements located around the PAS. Downstream elements include the U-rich and GU-rich elements, which are typically located within ∼100 nt downstream of the pA.

Most mammalian genes express alternative cleavage and polyadenylation (APA) isoforms [6], [7]. While the majority of alternative pAs are located in the 3′-most exon, leading to regulation of 3′ untranslated regions (3′UTRs), about half of the genes have pAs located in introns [8], leading to changes in coding sequences (CDSs) and 3′UTRs. Intronic pAs can be classified into two groups depending upon the splicing structure of the resultant terminal exon: composite terminal exon pA or skipped terminal exon pA. A composite terminal exon pA is located in a terminal exon which contains both exon and intron sequences. In this case, a 5′ splice site (5′SS) is located upstream of the pA. A skipped terminal exon pA is located in a terminal exon which can be entirely skipped in splicing. We previously found that composite terminal exon pAs in the human genome are typically located in large introns with weak 5′SS [9]. A classic model of composite terminal exon pA is the intronic pA of the immunoglobulin heavy chain M (IgM) gene [10]. IgM mRNAs switch from using a 3′-most exon pA to an intronic pA during activation of B cells, which results in a shift in protein production from a membrane-bound form to a secreted form.

In mammalian cells, over 20 proteins are directly involved in C/P [1], [11]. Some proteins form complexes, including the Cleavage and Polyadenylation Specificity Factor (CPSF), containing CPSF-160, CPSF-100, CPSF-73, CPSF-30, hFip1, and Wdr33; the Cleavage stimulation Factor (CstF), containing CstF-77, CstF-64, and CstF-50; Cleavage Factor I (CFI), containing CFI-68 or CFI-59 and CFI-25; and Cleavage Factor II (CFII), containing Pcf11 and Clp1. Single proteins involved in C/P include Symplekin, poly(A) polymerase (PAP), nuclear poly(A) binding protein (PABPN), and RNA Polymerase II (RNAPII). In addition, RBBP6, PP1α, PP1β are homologous to yeast C/P factors [12], whose functions in 3′ end processing are yet to be established in mammalian cells.

CstF-77 has been shown to interact with several proteins in the C/P complex, such as CstF-64 and CstF-50 in CstF [13], [14], [15], [16], CPSF-160 [17], and the carboxyl (C)-terminal domain (CTD) of RNAPII [18]. CstF-77 can dimerize through the second half of its amino (N)-terminal 12 HAT domains [15], [16], which is also responsible for dimerization of the CstF complex. Therefore, the role of CstF-77 in 3′ end processing appears to be bridging and/or positioning various factors for C/P. While CstF-77 has not been extensively studied in mammalian cells, its homolog in Drosophila, suppressor of forked or su(f), has been associated with a number of functions. First, su(f) regulates 3′ end processing of transposable elements, impacting their effects on cellular genes [14], [19], [20]; Second, su(f) regulates the usage of an intronic pA of its own pre-mRNA, creating an autoregulatory mechanism [21]; Third, su(f) is more expressed in mitotically active cells, which was suggested to be attributable to weak autoregulation in dividing cells compared to non-dividing ones [22], and the su(f) mutant strain showed a defect in cell proliferation [23]. However, not all parts of su(f) can be replaced by human CstF-77 for functional complementation, indicating structural and functional differences between these two proteins [24].

We previously identified a conserved pA in intron 3 (In3) of the human CstF-77 gene [25]. Here, we analyze the function of In3 pA and the significance of its flanking splicing and C/P features. We elucidate how In3 pA usage is related to global 3′UTR regulation across cells and tissues, and how In3 pA is regulated by C/P and splicing activities. We demonstrate that perturbation of CstF-77 expression leads to widespread APA and disturbance of cell proliferation and differentiation.

## Results

### The intronic pA of human CstF-77 gene is surrounded by various conserved features

**Figure 1:**
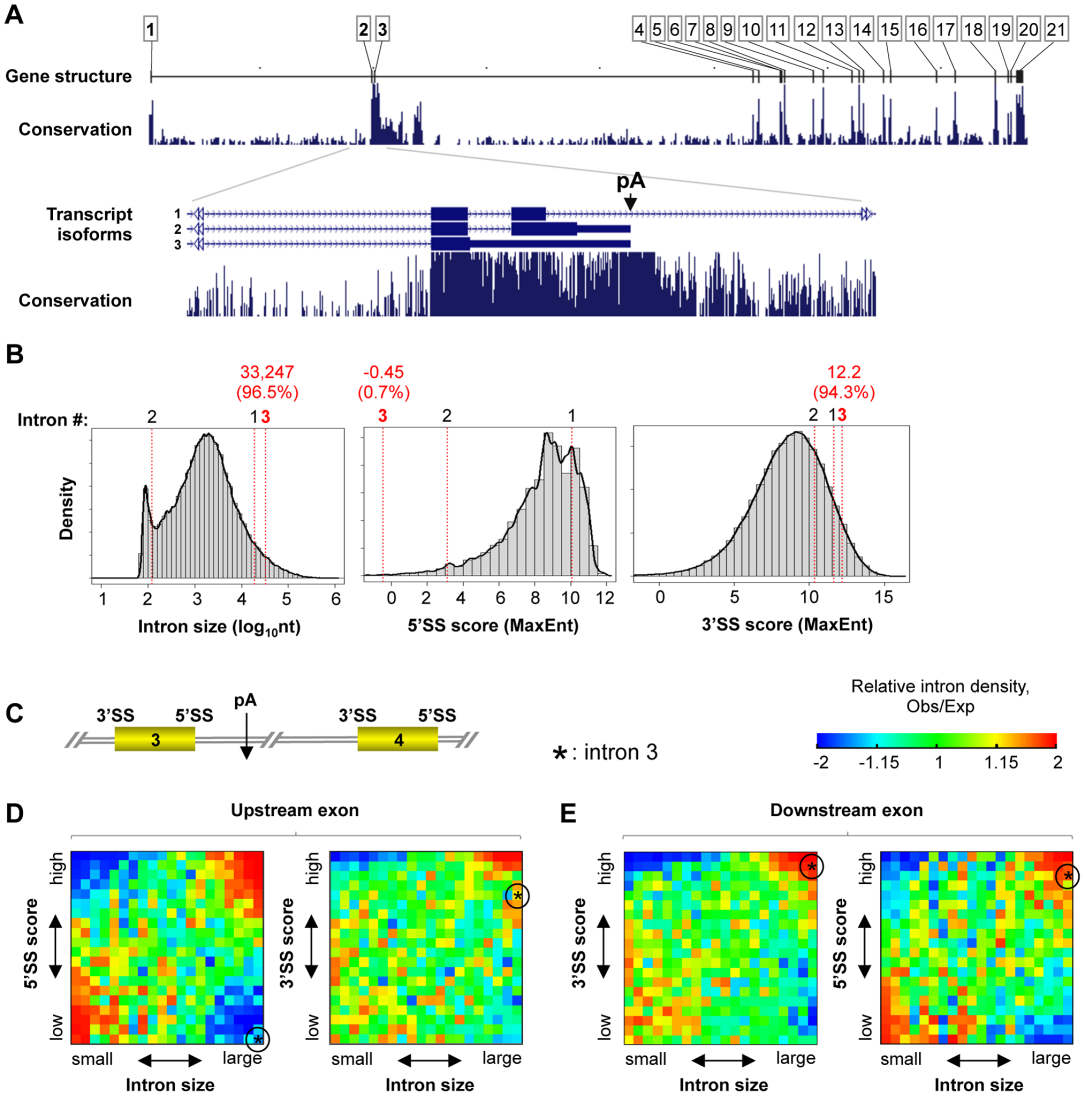
The intron 3 of human CstF-77 gene (*CTSF3*) has several unique, conserved features. (**A**) Gene structure of *CSTF3*, encoding the C/P factor CstF-77. Exons are numbered. A pA in intron 3 leads to APA isoforms 2 and 3 (isoform 3 has retention of intron 2). The conservation profile is based on vertebrate genomes. (**B**) Histograms of intron size, 5′SS maximum entropy (MaxEnt) score, and 3′SS MaxEnt score for all RefSeq-supported introns in the human genome. Red lines indicate introns 1–3 of *CSTF3*. For each feature of intron 3, its value and percent of introns with a lower value are indicated. (**C**) Schematic of intron 3 flanked by exons 3 and 4. (**D**) Density maps of introns in the human genome based on the 5′SS or 3′SS score of upstream exon and intron size. Intron density map is a heatmap showing distribution of introns with respect to two parameters (x-axis and y-axis). Left, 5′SS vs. intron size; right, 3′SS vs. intron size. Relative density is based on ratio of observed values to expected values (see Materials and Methods for detail), and is represented by colors using the color scale shown on the top. (**E**) As in (D), except that downstream 5′SS and 3′SS scores were used. The intron 3 of *CSTF3* is indicated by a circled asterisk in each graph.

We previously found that vertebrate genes encoding the C/P factor CstF-77 contain a conserved intronic pA (Figure S1) [25]. To elucidate the function of this pA, we first focused on the human CstF-77 gene (*CSTF3*), which has 21 exons (Figure 1A) with the conserved intronic pA located in intron 3 (In3). Remarkably, the 5′ portion of the gene before exon 4 accounts for 69% of the gene region. Both introns 1 and 3 are very large, with intron 3 (33.2 kb) being larger than 96% of all introns in the human genome and accounting for 43% of the gene region, whereas intron 2 is small, below 8% of all introns in the genome (Figure 1B, left panel). In addition, introns 1–3 are highly conserved in size across vertebrate CstF-77 genes, both in absolute and relative values (Figure S2A), suggesting functional relevance. Using the maximum entropy (MaxEnt) method to examine splice site strength [26], we found that the 5′ splice site (5′SS) of intron 3 is very weak, at the 0.7^th^-percentile of all introns in the human genome (Figure 1B, middle panel), whereas the 3′SS of intron 3 is very strong, at the 94.3^th^-percentile of all human introns (Figure 1B, right panel). Notably, using PhastCons scores [27], we found that the surrounding sequences of the 5′SS and intronic pA are much more conserved than those of other 5′SSs and pAs, respectively, in the human genome (Figure S2B). By contrast, conservation of sequence around the 3′SS is modest (Figure S2B).

We next examined how unique is the combination of large intron, weak 5′SS and strong 3′SS in the human genome. Since splicing in higher species is typically governed by the exon-definition model [28], we also included 3′SS of exon 3 and 5′SS of exon 4 in the analysis (Figure 1C). Using the intron density map (see Materials and Methods for detail) to simultaneously interrogate intron size and 5′SS or 3′SS strength (Figures 1D and 1E), we found that large introns in the human genome in general are flanked by strong 5′SS and 3′SS of both upstream and downstream exons, as indicated by enrichment of introns with these features relative to introns with randomized size and 5′SS or 3′SS strengths (shown as observed (Obs)/expected (Exp)). This trend holds for intron 3 of *CSTF3* except for its 5′SS. Indeed, the combination of large intron with weak 5′SS is significantly depleted in the human genome (Figure 1D). Therefore, the combination of large size and weak 5′SS of intron 3 is rather unique for human introns.

### Perturbations of splicing or pA parameters impact intronic C/P

**Figure 2:**
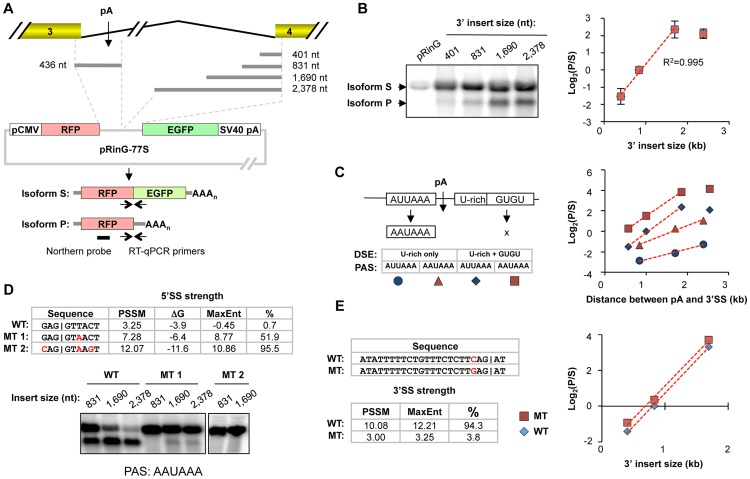
Contribution of intron size, 5′SS strength, 3′SS strength, and pA strength to the usage of the intronic pA of *CSTF3*. (**A**) Schematic of the pRinG-77S vector containing the 5′ region of intron 3 with the pA and variable 3′ regions of intron 3. Two isoforms (isoforms S and P) can be generated as indicated. Northern blot probe and RT-qPCR primers used to examine the expression of isoforms S and P are indicated. pCMV, CMV promoter; RFP, red florescent protein; EGFP, enhanced green florescent protein; SV40 pA, SV40 polyA site; AAA_n_, poly(A) tail. (**B**) Analysis of intron size. Left, Northern blot analysis of isoforms S and P expressed from the pRinG-77S vector containing different 3′ intron inserts as indicated in the graph; right, quantification of the Northern blot data. The R^2^ of linear regression is indicated. (**C**) Analysis of pA strength. Left, mutations of pA sequence and Northern blot analysis of isoforms; right, quantification of the Northern blot data. The pA represented by diamond is the wild type. The R^2^ values of linear regression lines are all >0.98. (**D**) Analysis of 5′SS strength. Top, wild type (WT) and mutant (MT) 5′SS sequences used in this study, with their strength analyzed by three methods, i.e., position specific scoring matrix (PSSM), ΔG for base pairing with U1 snRNA, and maximum entropy (MaxEnt). The percentile of the MaxEnt value based on all RefSeq-supported introns in the human genome is also shown. Bottom, Northern blot analysis of isoforms using different constructs. The constructs all have AAUAAA as PAS. (**E**) Analysis of 3′SS strength. Left, WT and MT 3′SS sequences and strength scores. The percentile value based on all RefSeq-supported introns in the human genome is also shown. Right, RT-qPCR analysis of isoforms S and P from the pRinG-77S vectors with WT or MT 3′SS. The R^2^ values of linear regression lines are >0.99. HeLa cells were used for all the Figure 2 studies.

To examine the significance of various features surrounding In3 pA of *CSTF3*, we constructed reporter plasmids (called pRinG-77S) containing an intron using the 5′SS and 3′SS of intron 3 (Figure 2A). The 5′ region also contained the In3 pA, which can lead to a short, intronic pA isoform (isoform P) encoding the red fluorescence protein (RFP). If the intronic pA is not used, a long, splicing isoform (isoform S) is expressed, which encodes both RFP and enhanced green fluorescence protein (EGFP)(Figure 2A).

To examine the importance of intron size, we cloned 3′ regions of intron 3 with various sizes. As the insert size increased, the amount of intronic pA product also increased (Figure 2B). A linear correlation between the ratio of isoform P to isoform S (log_2_), representing the intronic pA usage, and the insert size can be discerned for inserts from 401–1,690 nt. However, the ratio did not change when the insert size was increased to 2,378 nt. This regulation of intronic pA usage is due to the change of intron size rather than the distance between the intronic pA and the SV40 pA at the 3′ end of reporter gene, because no significant difference could be discerned when we expanded the region after 3′SS by adding another EGFP sequence (Figure S3A). In addition, a linear decrease of intronic pA usage was also observed when the distance between 5′SS and pA was expanded by random sequences (Figure S3B). Taken together, these data indicate that there is a kinetic competition between C/P and splicing in the usage of In3 pA.

Several cis elements around In3 pA are highly conserved across vertebrates, including the upstream UGUA and AUUAAA elements and downstream U-rich and GU-rich elements (Figure S1). To examine the contributions of these cis elements to the pA strength, we mutated AUUAAA to AAUAAA, a stronger C/P signal [29], and/or deleted the downstream GU-rich elements. We found that mutation of AUUAAA to AAUAAA led to an ∼2-fold increase in pA usage, whereas deletion of the GU-rich elements led to an ∼10-fold decrease (Figure 2C). Thus, this analysis indicates that the strength of In3 pA of *CSTF3* is at a suboptimal level. Interestingly, the slope of the curve for log_2_(P/S) vs. pA to 3′SS distance appeared different for constructs with GU-rich elements compared to those without (Figure 2C). By contrast, mutation of AAUAAA to AUUAAA did not lead to a slope change, suggesting that the contribution of GU-rich elements to pA strength may be different than that of PAS.

We next examined the importance of 5′SS and 3′SS strengths. We mutated the 5′SS sequence to two stronger sequences (mutants 1 and 2, Figure 2D) based on their MaxEnt scores. Mutants 1 and 2 would be at the 51.9^th^- and 95.5^th^-percentile, respectively, in the human genome. The relative strengths of the 5′SSs were also confirmed by comparison with the consensus sequence of all human 5′SSs, represented by position-specific scoring matrix (PSSM) scores, and by free energy values for base-pairing with U1 snRNA (Figure 2D). Strengthening 5′SS drastically inhibited intronic pA usage: ∼90% decrease for mutant 1 and no detectable intronic pA usage for mutant 2 even though AAUAAA was used as PAS (Figure 2D). We also weakened 3′SS strength from the 94.3^th^-percentile to the 3.8^th^-percentile based on the MaxEnt score. However, only a minor increase of intronic pA usage was detected (Figure 2E). Together, these data indicate 5′SS strength is a determining factor for the usage of In3 pA.

### Intronic pA usage is part of a feedback mechanism to regulation CstF-77 expression

In both human and mouse cells, the In3 pA can lead to 2 short isoforms (isoforms 2 and 3 in Figure 1A), depending upon whether or not intron 2 is spliced. The isoform 2, which does not have retention of intron 2, is ∼2–3-fold more abundant than isoform 3 in HeLa and C2C12 cells based on the semi-quantitative reverse transcription (RT)-PCR analysis (Figure S4). According to the open reading frames, isoform 2 would encode a protein of 103 amino acids (aa), containing the N-terminal region of CstF-77 and some aa from the intronic region of intron 3 (Figure S5), whereas isoform 3 would encode a protein of 44 aa. However, we could not detect these protein products using various antibodies against the N-terminal region of CstF-77. In addition, several lines of evidence indicate that the coding region from intron 3 may cause rapid degradation of the protein encoded in isoform 2: 1) pRinG-77S constructs expressing different amounts of intronic pA isoforms showed the same red fluorescence to green fluorescence ratio when transfected into HeLa cells (Figure S6A); 2) immunoblot analysis using an antibody against RFP did not detect protein products of the intronic isoforms expressed from pRinG-77S constructs (Figure S6B); 3) a bicistronic mRNA containing RFP tagged with the intronic coding sequence between 5′SS and stop codon followed by IRES and EGFP resulted in green fluorescence only (Figure S6C). Thus, it appears that the protein products from intronic pA isoforms are expressed at very low levels at most.

**Figure 3:**
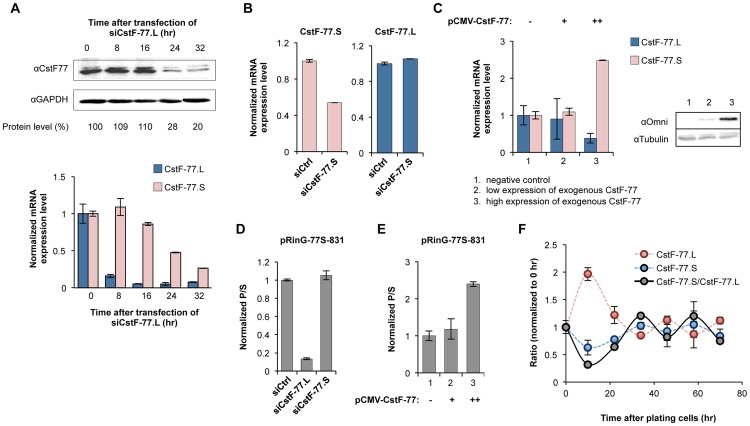
The intronic pA of *CSTF3* is involved in feedback autoregulation. (**A**) Effect of CstF-77 knockdown by siRNA on expression of CstF-77.L and CstF-77.S mRNAs. Top, time course of the protein level of CstF-77 after knockdown measured by Immunoblot (IB). Data were first normalized to GAPDH and then to the time 0 hr, as indicated below the image. Bottom, the effect of CstF-77 knockdown on the expression of CstF-77.L and CstF-77.S mRNAs. The time points are the same as those in (A). (**B**) Effect of CstF-77.S knockdown on expression of CstF-77.L. Left, knockdown of CstF-77.S as verified by qRT-PCR (both isoforms 2 and 3 are measured); right, effect of CstF-77.S knockdown on the expression of CstF-77.L. (**C**) Left, effect of CstF-77 overexpression on the expression of endogenous CstF-77.L and CstF-77.S mRNAs. Right, overexpression of exogenous CstF-77 as verified by IB using an antibody against the Omni tag of exogenous CstF-77. (**D**) Effect of CstF-77.L and CstF-77.S knockdowns on the P/S ratio of transcripts expressed from the reporter plasmid pRinG-77S-831. (**E**) Effect of CstF-77 overexpression on the P/S ratio of transcripts expressed from the reporter plasmid pRinG-77S-831. The expression levels of exogenous CstF-77 are the same as those in (C). (**F**) Time course for expression of endogenous CstF-77.L and CstF-77.S mRNAs and the CstF-77.S/CstF-77.L ratio after plating cells. All values were normalized to those at the 0 hr. All data on mRNA expression were based on RT-qPCR.

Given that CstF-77 is a C/P factor, we next reasoned that it may regulate the usage of its own intronic pA, creating a feedback autoregulatory mechanism, similar to its fly homolog su(f) [21]. To this end, we used small interfering RNAs (siRNAs) to specifically knock down the expression of the CstF-77 transcripts encoding full length protein (named CstF-77.L mRNAs). The CstF-77.L mRNA level significantly decreased after 8 hr of siRNA transfection and its protein level started to decrease after 16 hr (Figure 3A). Interestingly, expression of isoforms 2 and 3, collectively named CstF-77.S mRNAs, also gradually decreased after 16 hr, indicating that the expression of CstF-77.S mRNAs can be controlled by the CstF-77 protein level. By contrast, knockdown of CstF-77.S mRNAs (by ∼50%, Figure 3B, left) did not affect CstF-77.L mRNAs (Figure 3B, right), suggesting that expression of CstF-77.S mRNA is not important for CstF-77.L expression. In accord with the autoregulatory mechanism, expression of exogenous CstF-77 led to increased expression of endogenous CstF-77.S mRNAs and decreased expression of endogenous CstF-77.L mRNAs (Figure 3C). Consistently, knockdown of CstF-77.L mRNAs inhibited intronic pA usage for the reporter construct pRinG-77S-831 (structure shown in Figure 2A), whereas knockdown of CstF-77.S mRNAs had no effect (Figure 3D); and overexpression of CstF-77 enhanced intronic pA usage for the reporter construct (Figure 3E).

**Figure 4:**
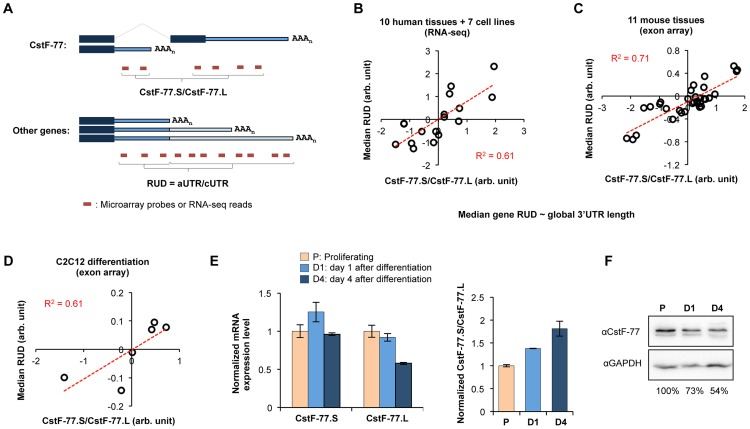
Intronic C/P of human and mouse CstF-77 genes correlates with the global 3′UTR length. (**A**) Analysis of the CstF-77.S/CstF-77.L ratio and global 3′UTR length regulation (RUD) using microarray and RNA-seq data. cUTR, constitutive UTR; aUTR, alternative UTR. Correlation of the CstF-77.S/CstF-77.L ratio with global 3′UTR length was found in 17 human tissues and cell lines (**B**), 11 mouse tissues (**C**), and proliferating and differentiating C2C12 cells (**D**) as indicated by the R^2^ values (linear regression). (**E**) Validation of CstF-77.S and CstF-77.L expression in proliferating and differentiating C2C12 cells by RT-qPCR (left). The normalized CstF-77.S/CstF-77.L ratio is also shown (right). P, proliferating; D1, differentiation for 1 day; D4, differentiation for 4 days. (**F**) Expression of CstF-77 protein in proliferating and differentiating C2C12 cells. The relative amount of CstF-77 is indicated. GAPDH was used as internal control.

We next reasoned that the negative feedback autoregulatory control may cause CstF-77.S and CstF-77.L isoforms to oscillate in their expression. To test this hypothesis, we examined expression of CstF-77.S and CstF-77.L mRNAs over time after plating cells. Indeed, as shown in Figure 4F, these two isoforms oscillated over time: when CstF77.L level was high CstF77.S level was low, and vice versa. Taken together, these data indicate that intronic pA usage is responsive to CstF-77 expression, creating a feedback autoregulatory mechanism.

### Global correlation between intronic pA usage of CstF-77 gene and APA of 3′UTRs

We previously found that the expression of C/P factors negatively correlates with the 3′UTR length in development and cell differentiation [30]. We asked whether the CstF-77.S/CstF-77.L ratio is related to APA of 3′UTRs. To this end, we analyzed an exon array dataset for 11 mouse tissues and a deep sequencing dataset for 10 human tissues and 7 human cell lines. The CstF-77.S/CstF-77.L ratio was calculated by comparing the intensity of microarray probes or density of RNA-seq reads for CstF-77.S with those for CstF-77.L (Figure 4A). The global 3′UTR length changes were calculated by comparing the intensity of microarray probes or density of RNA-seq reads for the region upstream of first pA in 3′UTR (called constitutive 3′UTR or cUTR) with those for the downstream region (called alternative 3′UTR or aUTR)(Figure 4A). This value was also called Relative expression of isoforms Using Distal pAs (RUD, see Materials and Methods for detail). The median RUD of all genes reflects the relative global 3′UTR length. Interestingly, the CstF-77.S/CstF-77.L ratio generally correlated with the global 3′UTR length in both human (R^2 = ^0.61, Pearson Correlation) (Figure 4B) and mouse cells/tissues (R^2 = ^0.71, Pearson Correlation)(Figure 4C). This result indicates that the CstF-77.S/CstF-77.L ratio is associated with APA of 3′UTRs.

We next analyzed our previously published exon array data for differentiation of C2C12 myoblast cells [31], with which we reported general lengthening of 3′UTR during cell differentiation. A linear correlation (R^2 = ^0.61) between CstF-77.S/CstF-77.L and RUD was also detected (Figure 4D). To validate this finding, we examined expression of CstF-77.S and CstF-77.L mRNAs by RT-qPCR in proliferating C2C12 cells and cells after 1 day or 4 days of differentiation. CstF-77.S mRNAs showed increased expression by ∼20% after 1 day of differentiation but no significant change of expression after 4 days. By contrast, the expression of CstF-77.L mRNAs gradually decreased (Figure 4E). Consequently, the CstF-77.S/CstF-77.L ratio gradually increased in differentiation (Figure 4E). Consistently, the CstF-77 protein level decreased by 27% after 1 day and by 46% after 4 days (Figure 4F). Thus, the usage of In3 pA of CstF-77 gene inversely correlates with CstF-77 protein level in cell differentiation. Given CstF-77's role in C/P, this result suggests that CstF-77 protein level may be the underlying reason for the connection between the CstF-77.S/CstF-77.L ratio and global 3′UTR length.

### Intronic pA usage is regulated by U1 snRNP

**Figure 5:**
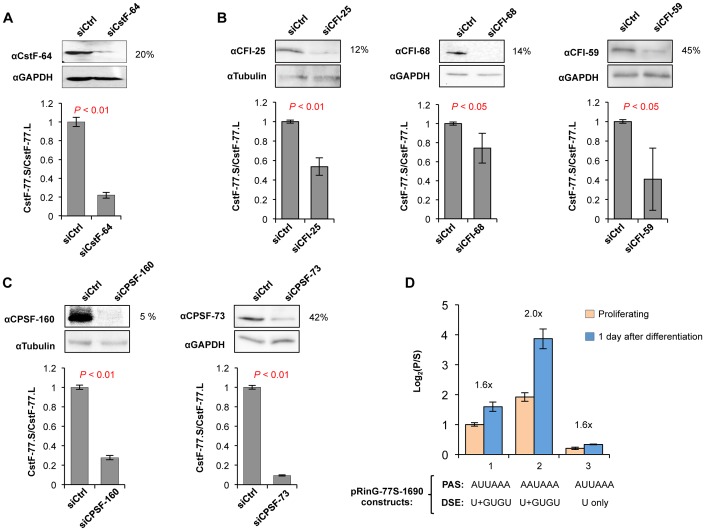
The intronic pA of CstF-77 gene is regulated by other C/P factors. (**A**) Effect of CstF-64 knockdown by siRNA on the CstF-77.S/CstF-77.L ratio. Top, Immunoblot (IB) showing knockdown efficiency; bottom, CstF-77.S/CstF-77.L ratio as measured by RT-qPCR. P-value (T-test) comparing knockdown and control siRNA samples is shown. (**B**) As in (A), except that knockdowns of CFI-25 (left), CFI-68 (middle), and CFI-59 (right) are shown. (**C**) As in (A), expect that knockdowns of CPSF-160 (left) and CPSF-73 (right) are shown. (**D**) Expression of isoforms P and S from various pRinG-77S-1690 reporter constructs in proliferating and differentiating (1 day of differentiation) cells. The PAS type and DSE type of each construct are indicated (see Figure 2C for detail). The ratio of the log_2_(P/S) value from proliferating cells to that from differentiating cells is indicated, where P represents the abundance of isoform P and S the abundance of isoform S. The difference in log_2_(P/S) value (proliferating cells vs. differentiating cells) is statistically significant (*P*<0.05, T-test) for all three constructs.

The increased CstF-77.S/CstF-77.L ratio in differentiation could be due to activation of C/P at In3 pA, which, however, seems in discord with our previous finding that the C/P activity in general is weakened in C2C12 differentiation [31]. Notably, intronic pA of CstF-77 without flanking 5′SS and 3′SS was less used in differentiated cells compared to proliferating cells by reporter assays (Figure S7), suggesting that the pA usage per se is decreased in differentiation. To explore this issue further, we knocked down several factors in the C/P machinery, including CstF-64 in the CstF complex (Figure 5A), CFI-25, CFI-68, and CFI-59 in the CFI complex (Figures 5B), and CPSF-160 and CPSF-73 in the CPSF complex (Figure 5C). All the knockdowns led to significant decrease of the CstF-77.S/CstF-77.L ratio, indicating that the pA usage is responsive to perturbation of the C/P activity. Furthermore, changing the pA strength does not alter the trend of CstF-77.S/CstF-77.L ratio changes in differentiation (Figure 5D).

**Figure 6:**
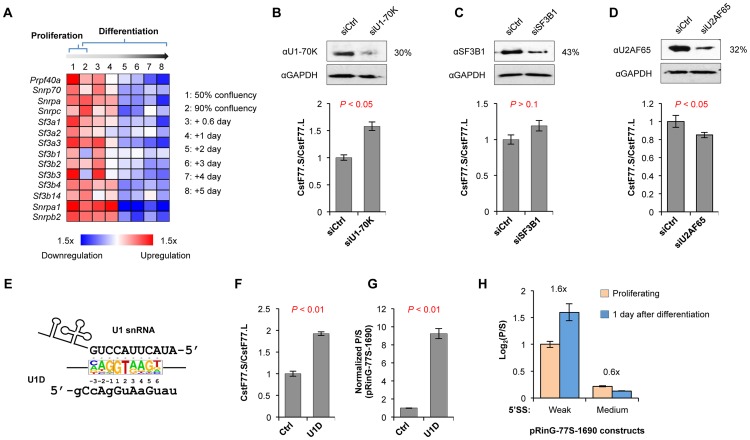
U1 snRNP regulates the usage of intronic pA of CstF-77 gene. (**A**) Regulation of expression of factors in U1 and U2 snRNPs in C2C12 differentiation. Expression analysis was based on a microarray dataset from the GEO database (GSE11415). Expression changes are represented by colors based on the scale shown at the bottom of the graph. (**B**) Effect of knockdown of U1-70K on the CstF-77.S/CstF-77.L ratio. Top, Immunoblot (IB) showing knockdown efficiency; bottom, CstF-77.S/CstF-77.L ratio as measured by RT-qPCR. P-value (T-test) comparing knockdown and control siRNA samples is shown. (**C**) and (**D**) As in (B), except that knockdowns of SF3B1 (C) and U2AF65 (D) are shown. (**E**) Inhibition of U1 snRNP interaction with 5′SS by U1D oligo. Sequence of the 5′ region of U1 snRNA is shown. The consensus sequence of the 5′SS of all RefSeq-supported human introns is represented by a sequence logo. U1D sequence is also shown (locked nucleic acid (LNA) residues are in uppercase and 2′-OMe RNA residues are in lowercase, see Materials and Methods for detail). (**F**) Effect of U1D on the CstF-77.S/CstF-77.L ratio. (**G**) Effect of U1D on the (isoform P)/(isoform S) ratio for pRinG-77S-1690. (**H**) The (isoform P)/(isoform S) ratios for pRinG-77S-1690 vectors with weak or medium strength 5′SS in proliferating and differentiating (1 day of differentiation) cells. See Figure 2D for 5′SS sequences. The ratio of the log_2_(P/S) value from proliferating cells to that from differentiating cells is indicated, where P represents the abundance of isoform P and S the abundance of isoform S. The difference in log_2_(P/S) value (proliferating cells vs. differentiating cells) is statistically significant (*P*<0.05, T-test) for all two constructs.

We next reasoned that since intron size and 5′SS strength can regulate the usage of intronic pA, change of splicing activity in differentiation may lead to change of CstF-77.S/CstF-77.L. Notably, mRNAs encoding several U1 snRNP and U2 snRNP factors are downregulated in differentiation based on microarray analysis (Figure 6A), suggesting weakening of their activities. To examine the effect of splicing regulation on CstF-77.S/CstF-77.L, we knocked down U1-70K, one of the components of U1 snRNP [32], SF3B1, a key component of U2 snRNP [33], and U2AF65, a factor involved in recognition of 3′SS and recruitment of U2 snRNP [34]. Knockdown of U1-70K led to a significant increase (∼50%) of the CstF-77.S/CstF-77.L ratio (*P*<0.05, Figure 6B), whereas knockdown of SF3B1 led to a marginal increase of the ratio (*P*>0.1, Figure 6C), and knockdown of U2AF65 led to a significant decrease of the ratio (*P*<0.05, Figure 6D). Thus, U1 snRNP may play a role in regulation of intronic pA usage in C2C12 differentiation.

To further explore the role of U1 snRNP in intronic C/P of CstF-77, we used an oligonucleotide which mimics the consensus sequence of 5′SS [35], termed U1 domain (U1D) oligo (Figure 6E). Presumably, U1D can sequester U1 snRNP in the cell, thereby inhibiting 5′SS recognition by U1 snRNP [35]. Upon treatment of U1D, the CstF-77.S/CstF-77.L ratio increased by 2-fold (Figure 6F). An even greater increase (∼9-fold) of intronic pA usage was observed from reporter assays using pRinG-77S-1690 (Figure 6G). Taken together, these results indicate that the intronic pA CstF-77 gene is under the control of U1 snRNP.

The effect of U1 snRNP regulation on intronic C/P is consistent with the critical role of 5′SS for pA usage (see above). To directly examine whether the 5′SS strength is important for intronic pA usage, we used reporter constructs with different 5′SS strengths in proliferating and differentiating C2C12 cells (Figure 6H). Interestingly, while the construct with wild type, weak 5′SS recapitulated the intronic pA usage of endogenous CstF-77 pre-mRNAs, the mutant 5′SS with medium strength showed the opposite trend, indicating that 5′SS strength is critical for the regulation of intronic pA usage. This result is in line with the general trend that intronic pAs activated in C2C12 differentiation tend to be in introns with weak 5′SS (Figure S8).

### CstF-77 plays an important role in cell proliferation and differentiation

**Figure 7:**
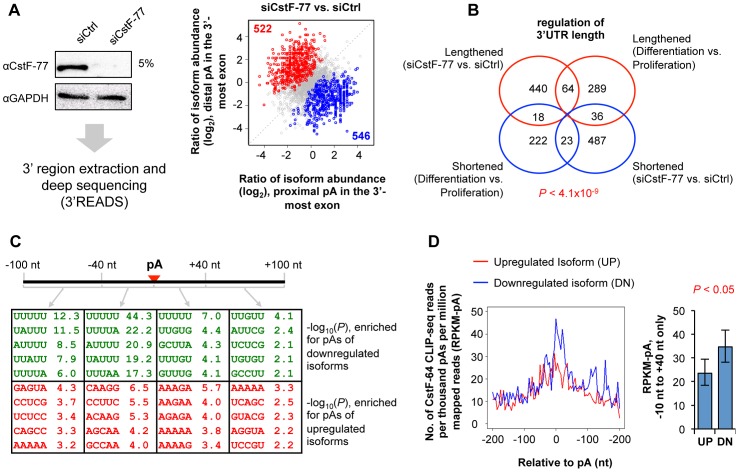
Knockdown of CstF-77 leads to widespread APA events. (**A**) Analysis of effect of CstF-77 knockdown in proliferating C2C12 cells by 3′READS. Left, IB of CstF-77 knockdown; right, regulation of alternative pAs in the 3′-most exon after CstF-77 knockdown. Genes with significantly upregulated distal (red) and proximal (blue) pA isoforms are indicated in each graph along with total numbers. Significantly regulated isoforms are those with *P*<0.05 (Fisher's exact test) and abundance change >5%. Only the two most abundant isoforms for each gene were analyzed. (**B**) Comparison of genes with 3′UTR changes between CstF-77 knockdown and C1C12 differentiation. The number of consistently regulated genes is significantly greater (*P* = 4.1×10^−9^, Chi-squared test) than the number of oppositely regulated ones. (**C**) Pentamers enriched for pAs of downregulated isoforms (top) and of upregulated isoforms (bottom). (**D**) CstF-64 binding around the pAs of upregulated isoforms (UP) and downregulated isoforms (DN). Left, binding profiles representing the number of CstF-64 CLIP-seq reads per thousand pAs per million mapped reads; right, aggregated reads in the −10 nt to +40 nt region around the pA. The difference between UP and DN groups is significant (*P*<0.05, bootstrap analysis).

In order to understand how critical it is to control CstF-77 expression in cell proliferation and differentiation, we knocked down CstF-77 in proliferating C2C12 cells and examined APA and gene expression genome-wide using our newly developed method, 3′ region extraction and deep sequencing (3′READS)(Figure 7A) [8]. We found 1,068 genes that had significant APA changes in 3′UTRs (*P*<0.05, Fisher's Exact test, and >5% change in isoform abundance)(Figure 7A). However, there was no significant bias of expression to proximal or distal pA isoforms, indicating no global 3′UTR shortening or lengthening after CstF-77 knockdown. Gene Ontology analysis indicated that genes with different functions were affected differently (Table 1). For example, genes with functions in “protein localization”, “intracellular transport”, “RNA processing” were more likely to have 3′UTRs lengthened, whereas those with functions in “cell-cell adhesion”, “mitosis”, and “Ras protein signal transduction” were more likely to have 3′UTRs shortened.

**Table 1:** Gene Ontology terms enriched for genes with regulated 3′UTRs after CstF-77 knockdown.

GO ID	GO Name	P-value
**Enriched for genes showing 3′UTR lengthening**		
GO:0008104	protein localization	7.1E-05
GO:0046907	intracellular transport	1.1E-04
GO:0006396	RNA processing	1.4E-04
GO:0022613	ribonucleoprotein complex biogenesis	3.4E-04
GO:0033554	cellular response to stress	6.4E-04
GO:0045454	cell redox homeostasis	1.4E-03
GO:0006457	protein folding	2.3E-03
GO:0006091	generation of precursor metabolites and energy	5.0E-03
GO:0019725	cellular homeostasis	5.7E-03
GO:0006412	translation	6.1E-03
**Enriched for genes showing 3′UTR shortening**		
GO:0007156	homophilic cell adhesion	3.0E-05
GO:0016337	cell-cell adhesion	4.6E-04
GO:0007067	mitosis	1.2E-03
GO:0007265	Ras protein signal transduction	1.4E-03
GO:0048285	organelle fission	1.6E-03
GO:0015031	protein transport	2.0E-03
GO:0007155	cell adhesion	3.1E-03
GO:0007049	cell cycle	5.3E-03

GO terms in the Biological Process category with *P*<0.001 are shown. Redundant GO terms (more than 70% overlap in associated genes with another GO term having a more significant *P*) are not shown.

We next compared this data with our recently published data for APA regulation in C2C12 differentiation [8]. Whereas only a small set of genes were found to be commonly regulated between CstF-77 knockdown and C2C12 differentiation, the number of consistently regulated genes was significantly greater than that of oppositely regulated genes (*P* = 4.1×10^−9^, Chi-squared test), suggesting downregulation of CstF-77 is involved in regulation of a subset of APA events in C2C12 differentiation (Figure 7B).

We next examined cis elements surrounding pAs of regulated isoforms. Remarkably, U-rich elements were significantly enriched for pAs whose isoforms were downregulated after CstF-77 knockdown, particularly in the −40 nt to −1 nt region relative to the pA (set to 0) (Figure 7C). This result suggests that pAs with U-rich elements are highly dependent on CstF-77 for C/P. Since CstF-77 is in the same complex as CstF-64, we next examined CstF-64 binding near regulated pAs using the CstF-64 CLIP-seq data we recently published [8]. Consistent with the interaction between CstF-77 and CstF-64, pAs of downregulated isoforms had significantly more CstF-64 binding in nearby regions than those of upregulated ones (*P*<0.05, bootstrap analysis), suggesting that usage of these pAs are also dependent on CstF-64 (Figure 7D).

**Figure 8:**
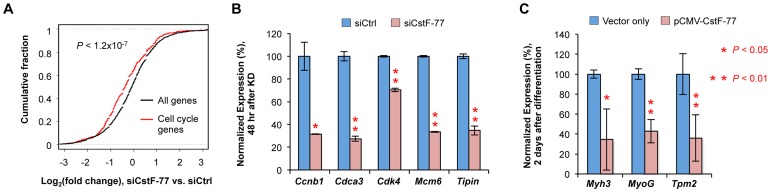
Cell cycle genes are affected by CstF-77 knockdown. (**A**) Cumulative fraction for gene expression changes (siCstF-77 vs. siCtrl) of all genes analyzed (11,396 in total) or cell cycle genes (540 in total). (**B**) Validation of mRNA expression changes by RT-qPCR for several cell cycle-related genes after CstF-77 knockdown. P-values (T-test) are indicated. (**C**) mRNA expression of C2C12 differentiation marker genes in cells over-expressing CstF-77 after 2 days of differentiation. Values were normalized to that of vector only. P-values are based on the T-test.

Our data also indicated that a large number of genes (1,776 in total) had significant changes of expression (fold change >1.5 and *P*<0.01, Fisher's Exact test) after CstF-77 knockdown. By GO analysis, we found, to our surprise, that genes related to cell cycle were most significantly downregulated (Table 2 and Figure 8A). This result was validated by RT-qPCR for a set of cell cycle-related genes, such as *Ccnb1* (cyclin B1), *Cdca3* (cell division cycle associated 3), *Cdk4* (cyclin-dependent kinase 4), *Mcm6* (minichromosome maintenance complex component 6), and *Tipin* (timeless interacting protein)(Figure 8B). This result indicates that the CstF-77 level is important for expression of cell cycle genes, and suggests that downregulation of CstF-77 may help cells halt proliferation and launch differentiation. To explore this further, we overexpressed CstF-77 in proliferating C2C12 cells, induced differentiation, and examined marker genes that are normally upregulated during differentiation. All three marker gene mRNAs, including *Myh3* (heavy polypeptide 3, skeletal muscle, embryonic), *MyoG* (myogenin), and *Tpm2* (tropomyosin 2, beta), were significantly less upregulated in cells overexpressing CstF-77 (Figure 8C), further indicating that the CstF-77 level is important for cell proliferation/differentiation.

**Table 2:** Gene Ontology terms enriched for upregulated and downregulated genes after CstF-77 knockdown.

GO ID	GO Name	P-value
**Enriched for downregulated genes**		
GO:0000278	mitotic cell cycle	5.4E-10
GO:0048285	organelle fission	1.4E-09
GO:0022403	cell cycle phase	2.2E-09
GO:0007049	cell cycle	3.5E-09
GO:0006396	RNA processing	1.6E-05
GO:0006259	DNA metabolic process	5.0E-05
GO:0051276	chromosome organization	8.2E-05
GO:0016337	cell-cell adhesion	4.2E-04
**Enriched for upregulated genes**		
GO:0030163	protein catabolic process	9.3E-07
GO:0009057	macromolecule catabolic process	3.1E-06
GO:0015031	protein transport	1.3E-05
GO:0006979	response to oxidative stress	1.3E-04
GO:0016265	death	5.2E-04

GO terms in the Biological Process category with *P*<0.001 are shown. Redundant GO terms (more than 75% overlap in associated genes with GO terms having a more significant *P*) are not shown.

## Discussion

In this study, we examined the evolution and regulation of intronic C/P of human and mouse CstF-77 genes. The conservation of various features involved in pA usage across vertebrates underscores its importance. Notably, the *Drosophila* gene encoding the homologue of CstF-77, *su(f)*, also contains an intronic pA [19]. Unlike the intronic pA isoforms of vertebrate CstF-77 genes, which have open reading frames, the *su(f)* intronic pA isoform does not have an in-frame stop codon. However, both vertebrate and fly intronic pAs appear to function to attenuate expression of the gene via feedback autoregulation. Remarkably, there is no conservation in surrounding sequences or adjacent intron/exon structures between the intronic pAs in vertebrates and in fly, indicating convergent evolution of this mechanism. Intriguingly, we could not find a similar mechanism in *C. elegans* after exhaustive search of all available public pA data. It remains to be seen whether or not the CstF-77 homolog in *C. elegans* is subject to another type of autoregulation.

In addition to autoregulation, we found that the intronic pA usage is regulated upon perturbation of several other C/P factors, including those in the CstF, CPSF and CFI complexes, suggesting it is responsive to the general C/P activity in the cell. Two key features of the intronic pA of the CstF-77 gene may make it particularly suitable for this function: first, its suboptimal strength can create a wide dynamic range of usage in response to change of C/P activity; second, its placement in an intron can allow rapid regulation because of competition of its usage with splicing.

**Figure 9:**
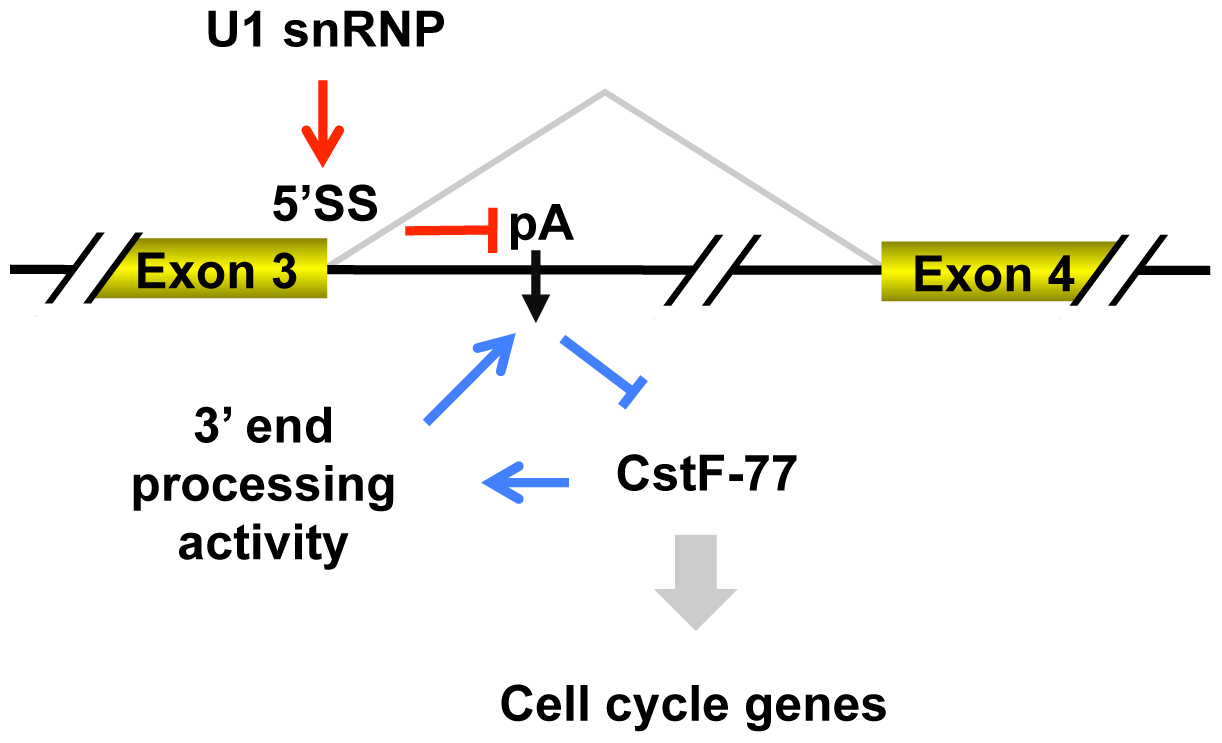
Model for regulation of intronic pA of the CstF-77 gene by 3′ end processing and U1 snRNP, and its impact on expression of cell cycle genes.

Juge et al. proposed two modes of autoregulation for fly su(f), a strong mode in non-dividing cells and a weak mode in dividing cells [22]. Here, our study indicates that splicing plays a dominant role in the usage of intronic pA of CstF-77 gene. Consistently, inhibition of the U1 snRNP activity, not the C/P activity, recapitulates the intronic pA regulation in cell differentiation. Thus, we propose that the U1 snRNP activity sets the general level of intronic pA usage under different conditions, such as in cell proliferation and differentiation, and the C/P activity plays a fine tuning role to robustly control CstF-77 expression under a given condition (Figure 9). This model would readily explain the two modes of autoregulation proposed by Juge et al., i.e., the U1 snRNP activity is high in dividing cells and weak in non-dividing cells. Moreover, control of CstF-77 level by U1 snRNP suggests that the C/P activity in the cell is modulated by the splicing activity, leading to coordination between these two pre-mRNA processing steps. This coordination may ensure that the widespread cryptic pAs in introns are not activated when U1 snRNP is downregulated under conditions like cell differentiation [9]. Conversely, this result may explain, at least partially, that mild inhibition of U1 snRNP can lead to 3′UTR lengthening [36].

Regulation of intronic pA of CstF-77 is reminiscent of a similar mechanism for the IgM gene. Both intron size and 5′SS strength were found to be important for the usage of intronic pA in the IgM gene [37], [38]. A number of factors have been implicated in the regulation, including the C/P factor CstF-64 [10] , the U1 snRNP component U1A [39], and the RNAPII transcription elongation factor ELL2 [40]. Whereas we found U1 snRNP regulation correlates with the activation of intronic pA of CstF-77 gene, future studies are needed to examine whether additional mechanisms can contribute to this regulation. Of particular importance is whether other splicing factors also play a role in the regulation of intronic pA. Notably, splicing factors in general are downregulated in C2C12 differentiation (Figure S9). Here we observed only marginal activation of In3 pA after SF3B1 knockdown and, surprisingly, inhibition of the pA after U2AF65 knockdown. It remains to be seen how factors involved in different steps of splicing regulate the usage of In3 pA and intronic C/P in general.

Perturbation of CstF-77 expression led to widespread APA and expression changes of a large number of genes. Remarkably, the genes with functions in cell cycle are most significantly affected, indicating that they are highly dependent upon CstF-77 for expression. pAs surrounded with U-rich elements appeared to be more affected by CstF-77 knockdown. Indeed, we found downregulated genes with a single pA also tend to have U-rich elements surrounding the pA (Figure S10), suggesting that inefficient 3′ end processing may lead to their downregulation of expression. Intriguingly, cell cycle genes tend to have shortened 3′UTRs after CstF-77 knockdown (Table 1). Since genes with shortened 3′UTRs tend to be downregulated (Figure S11), it is possible that distal pAs of cell cycle genes are more responsive to the CstF-77 level. Future work is needed to fully unravel the mechanism by which CstF-77 regulates cell cycle genes.

We found pAs with more CstF-64 binding are more likely to be affected by CstF-77 knockdown indicating that some of the regulation is through the CstF complex. However, CstF-64 is also known to bind GU-rich elements [41], and our cross-linking immunoprecipitation and high-throughput sequencing (CLIP-seq) data from C2C12 cells showed that the top two most significant pentamers for CstF-64 binding are UGUGU and UUUUU [8]. But GU-rich elements are only modestly enriched in the downstream region of regulated pAs after CstF-77 knockdown. Whether pAs with a different number or placement of U-rich and UG-rich elements are differentially regulated by CstF-77 and CstF-64 needs to be examined in the future. Moreover, a recent genome-wide study of CstF-64 knockdown in HeLa cells indicated that only a small set of APA events in these cells are regulated by the factor [42]. However, co-depletion of CstF-64 and its paralog τCstF-64 leads to more APA changes, largely leading to 3′UTR lengthening. That APA pattern appears different than that observed in this study with CstF-77 knockdown. Whether the difference is due to different levels of knockdown or different cell types used in the studies needs to be further explored.

## Materials and Methods

### Plasmids

Construction of the pRinG vector and all plasmids derived from pRinG are described in Table S1. The pRiG vector and pRiG-77.AE containing the intronic pA of CstF-77 were described previously [31]. For pCMV-CstF-77, the open reading frame (ORF) of human CstF-77 was obtained from the IMAGE clone 5223351 (Invitrogen) by PCR using primers 5′-CGATGAATTCATGTC AGGAGACGGAGCC and 5′-GGCCCTCGAGCTACCGAATCCGCTTCTG. The fragment was digested by EcoR I and Xho I, and then inserted into the pcDNA3.1/His C vector (Invitrogen) digested with the same enzymes.

### Cell culture, differentiation, and transfection

HeLa cells and C2C12 cells were maintained in Dulbecco's Modified Eagles Medium (DMEM) supplemented with 10% fetal bovine serum (FBS). Differentiation of C2C12 cells was induced by switching cell media to DMEM+ 2% horse serum (Sigma) when cells were ∼100% confluent. All media were also supplemented with 100 units/ml penicillin and 100 µg/ml streptomycin. Transfection with plasmids or siRNAs was carried out with Lipofectamine™ 2000 (Invitrogen) or jetPEI(polyplus) according to manufacturer's recommendations. Transfection was carried out for 48 hr unless described otherwise. siRNA sequences are shown in Table S2. The U1D oligo (5′-gCcAgGuAaGuau) and control oligo (5′-CAGAAATACACAATA), where locked nucleic acid (LNA) residues are in uppercase, 2′-OMe RNA residues are in lowercase, DNA nucleotides are in underlined uppercase, were previously described in Goraczniak et al. [35] (called UA17-13B-U1D and UA17-13B-TD, respectively). These oligos were transfected into C2C12 cells at 15 µM using Lipofectamine 2000 when the confluency of cells was about 50%. Cells were harvested 48 hr after transfection.

### FACS and immunoblot

For fluorescent activated cell sorting (FACS) analysis, cells were released from culture dishes by Trypsin-EDTA 24 h after transfection and green and red fluorescence signals were read at 530 nm and 585 nm, respectively, in the BD FACScalibur system (BD Biosciences). For immunoblot, the RIPA buffer (1% NP-40, 0.1% SDS, 50 mM Tris-HCl pH 7.4, 150 mM NaCl, 0.5% Sodium Deoxycholate, and 1 mM EDTA) was used to extract proteins from the cell. Proteins were resolved by SDS-PAGE, followed by immunoblotting using antibodies. Antibodies used in this study and their sources are shown in Table S3.

### Northern blot and RT-qPCR

Total cellular RNA was extracted using Trizol (Invitrogen) according to manufacturer's protocol. RNA was run in a 1.2% denaturing agarose gel, and was transferred to nylon membrane overnight. RNA was detected by hybridization with a radioactively labeled probe for the RFP sequence. The probe was made by PCR using pDsRED-Express-c1 as template and primers 5′-CGATGCTAGCATGGCCTCCTCCGAGGAC and 5′-GGCCCTCGAGCTACAGGAACAG GTGGTG with α-^32^P-dCTP. For RT-qPCR, mRNA was reverse-transcribed using the oligo-dT primer (Promega), and qPCR was carried out with Syber-Green I as dye. Primers are shown in Table S4.

### Analysis of DNA microarray and RNA-seq data

To calculate the global 3′UTR length (RUD) score, we used exon array data for C2C12 cell differentiation [31], exon array data for mouse tissues (http://www.affymetrix.com/support/technical/sample_data/exon_array_data.affx), and RNA-seq data for human tissues and cell lines [7]. Exon array data were first normalized by the Robust Multichip Average (RMA) method. Expressed genes were selected by the Detection Above Background (DABG) method. The RUD score was based on the ratio of average probeset intensity of aUTR to that of cUTR, as previously described [31]. For RNA-seq data, the RUD score was based on the ratio of read density of aUTR to that of cUTR, as described previously [43]. For both analyses, aUTRs and cUTRs were defined by PolyA_DB 2 [44]. The relative expression of CstF-77.S vs. CstF-77.L was calculated based on the probes or RNA-seq reads specific for each isoform. For analysis of splicing factor expression, we examined mRNAs encoding splicing factors defined by Jurica and Moore [45].

### Analysis of introns

5′SS and 3′SS were analyzed as previously described [9]. Briefly, we used all GT-AT type introns supported by human RefSeq sequences to build Position Specific Scoring Matrices (PSSMs) for 5′SS and 3′SS. For 5′SS, we used −3 to +6 nt surrounding the 5′SS, with 3 nt in the exon and 6 nt in the intron; for 3′SS, we used −22 to +2 nt surrounding the 3′SS, with 22 nt in the intron and 2 nt in the exon. The maximum entropy scores were calculated with MaxEnt [26]. 5′SS sequences were also scored by their ability to anneal with U1 snRNA. We used the sequence 5′-ACUUACCUG of U1 snRNA to form duplex structures with 5′SS sequences using the RNAduplex function of ViennaRNA [46]. For intron density map, we used all the RefSeq-supported introns as the observed set, and created an expected set using randomized pairs of intron size and splice site. The introns were divided into 20 fractions based on intron size and splice site strength, respectively, and distributed in a 20×20 grid. For each cell in the grid, the ratio of the number of introns in the observed set to that in the expected set was calculated and represented by color in a heatmap.

### 3′READS

The 3′ region extraction and deep sequencing (3′READS) method used in this study is the same as previously described [8], except that 10 µg of total RNA was used, and poly(A)+ RNA was selected by the chimeric U_5_ and T_45_ (CU_5_T_45_) oligo conjugated on streptavidin beads and fragmented by 1 U RNase III at 37°C for 30 min. Poly(A)+ RNA fragments were subject to further processing as previously described [8]. pA identification was carried out as previously described [8]. Only poly(A) site supporting (PASS) reads, defined as having >2 non-genomic Ts at the beginning of read, were used for further analysis. The expression level of an APA isoform was calculated using the number of PASS reads assigned to the pA. To study APA in the 3′-most exon, we first selected the top two expressed isoforms and used the Fisher Exact test to examine their difference in abundance between CstF-77 knockdown and control. Significantly regulated isoforms were those with P<0.05 and change of abundance >5%. Gene expression was calculated using the total number of PASS reads assigned to a gene. Differentially regulated genes after CstF-77 knockdown were those with P<0.01 (Fisher's Exact test) and fold change >1.5 compared to control. The DAVID software was used to identify Gene Ontology terms enriched for genes with significant changes in gene expression or APA [47].

### *Cis*-element analysis

We examined four regions around the pA, i.e., −100 to −41 nt, −40 to −1 nt, +1 nt to +40 nt and +41 nt to +100 nt. For each region, the Fisher's Exact test was used to check whether a sequence was enriched for a set of pAs vs. another set, for example, those of upregulated isoforms vs. downregulated isoforms.

### CLIP-seq data analysis

CLIP-seq reads of CstF-64 [8] were aligned to the mouse genome (mm9) using Novoalign (http://www.novocraft.com/). Reads with deletions caused by skipping of reverse transcriptase at the UV cross-linked bases were used for analyses. A bootstrapping method [8] was used to compare CstF-64 binding in the −10 to +40 nt region around the pA for pAs of upregulated isoforms vs. those of downregulated isoforms after CstF-77 knockdown.

## Supporting Information

Figure S1 Alignment of vertebrate genomic sequences surrounding the intronic pA of CstF-77. Exon 3, intron 3, 5′SS, pA are indicated. Several conserved key cis elements are also indicated, including UGUA, PAS, U-rich, and GUGU elements. The stop codon for the isoform 2 of human CstF-77 is boxed. The 3′ end of pA with U-rich only DSE is indicated by an arrow.
(PDF)

Figure S2 Conservation analysis of intron 3 of *CSTF3*. (**A**) Conservation of intron size for introns 1, 2 and 3 of *CSTF3* across vertebrates. Left, intron size; right, fraction of introns smaller than the indicated intron in a specific species. (**B**) Conservation profiles around 5′SS (left), 3′SS (middle) and pA (right). PhastCons score distribution for each region was calculated based on 17 vertebrate species. Red lines represent *CSTF3* and black lines represent all other RefSeq-supported introns in the human genome or all other pAs in the human genome reported in PolyA_DB 2.
(PDF)

Figure S3 Analysis of the effect of the distance between the intronic pA and 3′-most pA or the 5′SS. (**A**) Left, Northern blot analysis of mRNA expressed from the pRinG-77S vectors containing an intronic insert of 831 nt and one or two EGFP sequences, as indicated in the graph; Right, quantification of the Northern blot data. (**B**) Left, two random sequences (354 nt and 649 nt) were inserted into the region between 5′SS and pA (indicated in the graph). Expression was analyzed by RT-qPCR using the indicated primer pairs. Right, quantification of isoform expression using RT-qPCR.
(PDF)

Figure S4 Analysis of isoforms 2 and 3 in HeLa and C2C12 cells. (**A**) Schematic showing primers used to examine isoforms 2 and 3. (**B**) RT-PCR products of isoforms 2 and 3 at different cycles of PCR. (**C**) Relative expression of isoforms 2 vs. 3 in HeLa and C2C12 cells. Quantification of expression was based on the RT-PCR products shown in (B).
(PDF)

Figure S5 Protein sequences encoded in CstF-77 mRNA isoforms. Protein motifs and domains are indicated.
(PDF)

Figure S6 The coding sequence of intron 3 inhibits protein expression. (**A**) Average red fluorescent intensity vs. average green fluorescent intensity for HeLa cells transfected with different pRinG-77S constructs (Left). Right, examples of FACS analysis result for pRinG-77S-401 and pRinG-77S-1690-AT. (**B**) Immunoblot (IB) with HeLa cells expressing different constructs using antibody against RFP. The expected size of the protein product expressed from the intronic pA isoform of pRinG-77S is ∼30 kDa. Note: the protein of ∼37 kDa in the pRinG-77S-401 lane is likely to be a degradation product of the RFP-EGFP fusion protein (major band at ∼56 kDa). (**C**) Top, schematic of pRiG-77Sin, which contains the intronic sequence from 5′SS to stop codon inserted into the pRiG vector. Bottom, FACS analysis of HeLa cells transfected with pRiG and pRiG-77Sin.
(PDF)

Figure S7 pA usage is inhibited in differentiating C2C12. Top, pRiG construct containing the intronic pA of CstF-77. Bottom, analysis of pA usage using pRiG.
(PDF)

Figure S8 Introns containing pAs upregulated in C2C12 differentiation tend to have a weak 5′SS. Boxplot of 5′SS strength (MaxEnt score) for introns containing upregulated pAs (red) and other introns containing pAs (grey).
(PDF)

Figure S9 Splicing factors are generally downregulated in C2C12 differentiation. Cumulative fraction lines for gene expression changes of splicing factor genes (172 in total) and of other genes are shown. P-value based on the Kolmogorov-Smirnov (KS) test is shown, which compares the distributions of two gene sets. The data were derived from the microarray dataset GSE11415 in the GEO database.
(PDF)

Figure S10 Pentamers enriched for pAs of downregulated (top) or upregulated genes (bottom). Only genes with a single pA were used for analysis.
(PDF)

Figure S11 Gene expression changes vs. 3′UTR regulation after CstF-77 knockdown. (**A**) All genes. (**B**) Cell cycle genes. For each gene set, genes were divided into 3 groups based on 3′UTR regulation. P-values (Wilcoxon test) comparing two sets are shown.
(PDF)

Table S1 Constructs used for reporter assays.
(DOCX)

Table S2 siRNAs used in this study. All siRNAs are for mouse genes unless indicated otherwise.
(DOCX)

Table S3 Antibodies used in this study.
(DOCX)

Table S4 RT-qPCR primers used in this study.
(DOCX)
