# Supplementary material for: The Conserved Intronic Cleavage and Polyadenylation Site of CstF-77 Gene Imparts Control of 3′ End Processing Activity through Feedback Autoregulation and by U1 snRNP
Source: PLoS Genet. 2013 Jul 11;9(7):e1003613. doi: 10.1371/journal.pgen.1003613 (PMC3708835; doi:10.1371/journal.pgen.1003613)
Supplement: Figure S1 — Alignment of vertebrate genomic sequences surrounding the intronic pA of CstF-77. Exon 3, intron 3, 5′SS, pA are indicated. Several conserved key cis elements are also indicated, including UGUA, PAS, U-rich, and GUGU elements. The stop codon for the isoform 2 of human CstF-77 is boxed. The 3′ end of pA with U-rich only DSE is indicated by an arrow. (PDF) [file pgen.1003613.s001.pdf]

# Figure S1

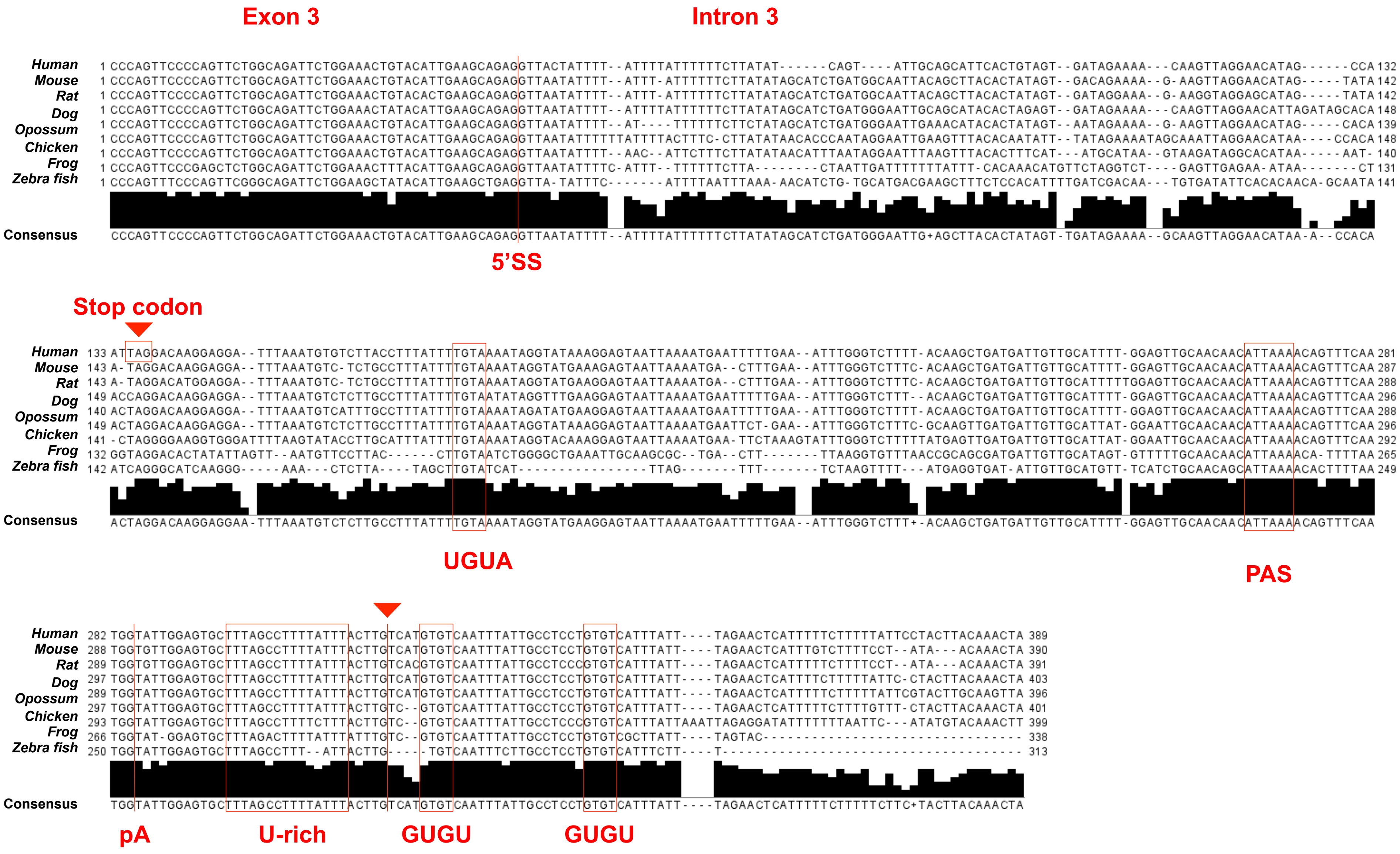
Exon 3
Intron 3
Human 1 CCCAGTTCCCCAGTTCTGGCAGATTCTGGAAACTGTACATTGAAGCAGAGGTTACTATTTT--ATTTTATTTTTTCTTATAT------CAGT----ATTGCAGCATTCACTGTAGT--GATAGAAAA---CAAGTTAGGAACATAG------CCA 132
Mouse 1 CCCAGTTCCCCAGTTCTGGCAGATTCTGGAAACTGTACATTGAAGCAGAGGTTAATATTTT--ATTT-ATTTTTTCTTATATAGCATCTGATGGCAATTACAGCTTACACTATAGT--GACAGAAAA--G-AAGTTAGGAACATAG-----TATA 142
Rat 1 CCCAGTTCCCCAGTTCTGGCAGATTCTGGAAACTGTACACTGAAGCAGAGGTTAATATTTT--ATTT-ATTTTTTCTTATATAGCATCTGATGGCAATTACAGCTTACACTATAGT--GATAGGAAA--G-AAGGTAGGAGCATAG-----TATA 142
Dog 1 CCCAGTTCCCCAGTTCTGGCAGATTCTGGAAACTATACATTGAAGCAGAGGTTAATATTTT--ATTTTATTTTTTCTTATATAGCATCTGATGGGAATTGCAGCATACACTAGAGT--GATAGAAAA---CAAGTTAGGAACATTAGATAGCACA 148
Opossum 1 CCCAGTTCCCCAGTTCTGGCAGATTCTGGAAACTGTACATTGAAGCAGAGGTTAATATTTT--AT----TTTTTTCTTCTATAGCATCTGATGGGAATTGAAACATACACTATAGT--AATAGAAAA--G-AAGTTAGGAACATAG-----CACA 139
Chicken 1 CCCAGTTCCCCAGTTCTGGCAGATTCTGGAAACTGTACATTGAAGCAGAGGTTAATATTTTTTATTTTACTTTC-CTTATATAACACCCAATAGGAATTGAAGTTTACACAATATT--TATAGAAAATAGCAAATTAGGAACATAA----CCACA 148
Frog 1 CCCAGTTCCCGAGCTCTGGCAGATTCTGGAAACTTTACATTGAAGCAGAGGTTAATATTTTC-ATTT--TTTTTTCTTA--------CTAATTGATTTTTTTATTT-CACAAACATGTTCTAGGTCT----GAGTTGAGAA-ATAA-------CT 131
Zebra fish 1 CCCAGTTTCCCAGTTCGGCAGATTCTGGAAGCTATACATTGAAGCTGAGGTTA-TATTTC------ATTTTAATTTAAA-AACATCTG-TGCATGACGAAGCTTTCTCCACATTTTGATCGACAA---TGTGATATTCACAACA-GCAATA 141
Consensus CCCAGTTCCCCAGTTCTGGCAGATTCTGGAAACTGTACATTGAAGCAGAGGTTAATATTTT--ATTTTATTTTTTCTTATATAGCATCTGATGGGAATTG+AGCTTACACTATAGT-TGATAGAAAA--GCAAGTTAGGAACATAA-A--CCACA
5’SS
Stop codon
Human 133 ATTAGGACAAGGAGGA--TTTAAATGTGTCTTACCTTTATTTTGTAAAATAGGTATAAAGGAGTAATTAAAATGAATTTTTGAA--ATTTGGGTCTTTT-ACAAGCTGATGATTGTTGCATTTT-GGAGTTGCAACAACATTAAAACAGTTTCAA 281
Mouse 143 A-TAGGACAAGGAGGA--TTTAAATGTC-TCTGCCTTTATTTTGTAAAATAGGTATGAAAGAGTAATTAAAATGA--CTTTGAA--ATTTGGGTCTTTC-ACAAGCTGATGATTGTTGCATTTT-GGAGTTGCAACAACATTAAAACAGTTTCAA 287
Rat 143 A-TAGGACATGGAGGA--TTTAAATGTC-TCTGCCTTTATTTTGTAAAATAGGTATGAAGGAGTAATTAAAATGA--CTTTGAA--ATTTGGGTCTTTC-ACAAGCTGATGATTGTTGCATTTTTGGAGTTGCAACAACATTAAAACAGTTTCAA 288
Dog 149 ACCAGGACAAGGAGGA--TTTAAATGTCTCTTGCCTTTATTTTGTAATATAGGTTTGAAGGAGTAATTAAAATGAATTTTTGAA--ATTTGGGTCTTT--ACAAGCTGATGATTGTTGCATTTT-GGAGTTGCAACAACATTAAAACAGTTTCAA 296
Opossum 140 ACTAGGACAAGGAGGA--TTTAAATGTCATTTGCCTTTATTTTGTAAAATAGATATGAAGGAGTAATTAAAATGAATTTTTGAA--ATTTGGGTCTTTT-ACAAGCTGATGATTGTTGCATTTT-GGAGTTGCAACAACATTAAAACAGTTTCAA 288
Chicken 149 ACTAGGACAAGGAGGA--TTTAAATGTCTCTTGCCTTTATTTTGTAAAATAGGTATGAAGGAGTAATTAAAATGAATTCT-GAA--ATTTGGGTCTTTC-GCAAGTTGATGATTGTTGCATTAT-GGAATTGCAACAACATTAAAACAGTTTCAA 296
Frog 141 -CTAGGGGAAGGTGGGATTTTAAGTATACCTTGCATTTATTTTGTAAAATAGGTACAAAGGAGTAATTAAAATGAA-TTCTAAAGTATTTGGGTCTTTTTATGAGTTGATGATTGTTGCATTAT-GGAATTGCAACAACATTAAAACAGTTTCAA 292
Zebra fish 132 GGTAGGACACTATATTAGTT--AATGTTCCTTAC------CTTGTAATCTGGGGCTGAAATTGCAAGCGC--TGA--CTT-------TTAAGGTGTTTAACCGCAGCGATGATTGTTGCATAGT-GTTTTTGCAACAACATTAAAACA-TTTTAA 265
142 ATCAGGGCATCAAGGG-----AAA---CTCTTA----TAGCTTGTATCAT---------------TTAG-------TTT-------TCTAAGTTTT---ATGAGGTGAT-ATTGTTGCATGTT-TCATCTGCAACAGCATTAAAACACTTTTAA 249
Consensus ACTAGGACAAGGAGGAA-TTTAAATGTCTCTTGCCTTTATTTTGTAAAATAGGTATGAAGGAGTAATTAAAATGAATTTTTGAA--ATTTGGGTCTTT+-ACAAGCTGATGATTGTTGCATTTT-GGAGTTGCAACAACATTAAAACAGTTTCAA
UGUA
PAS
Human 282 TGGTATTGGAGTGCTTTAGCCTTTTATTTACTTGTCATGTGTCAATTTATTGCCTCCTGTGTCATTTATT----TAGAACTCATTTTTCTTTTTATTCCTACTTACAAACTA 389
Mouse 288 TGGTGTTGGAGTGCTTTAGCCTTTTATTTACTTGTCATGTGTCAATTTATTGCCTCCTGTGTCATTTATT----TAGAACTCATTTGTCTTTTCCT--ATA---ACAAACTA 390
Rat 289 TGGTGTTGGAGTGCTTTAGCCTTTTATTTACTTGTCACGTGTCAATTTATTGCCTCCCGTGTCATTTATT----TAGAACTCATTTTTCTTTTCCT--ATA---ACAAACTA 391
Dog 297 TGGTATTGGAGTGCTTTAGCCTTTTATTTACTTGTCATGTGTCAATTTATTGCCTCCTGTGTCATTTATT----TAGAACTCATTTTCTTTTTATTC-CTACTTACAAACTA 403
Opossum 289 TGGTATTGGAGTGCTTTAGCCTTTTATTTACTTGTCATGTGTCAATTTATTGCCTCCTGTGTCATTTATT----TAGAACTCATTTTTCTTTTTATTCGTACTTGCAAGTTA 396
Chicken 297 TGGTATTGGAGTGCTTTAGCCTTTTATTTACTTGTC--GTGTCAATTTATTGCCTCCTGTGTCATTTATT----TAGAACTCATTTTTCTTTTGTTT-CTACTTACAAACTA 401
Frog 293 TGGTATTGGAGTGCTTTAGCCTTTTCTTTACTTGTC--GTGTCAATTTATTGCCTCCCGTGTCATTTATTAAATTAGAGGATATTTTTTTAATTC---ATATGTACAAACTT 399
Zebra fish 266 TGGTAT-GGAGTGCTTTAGACTTTTATTTATTTGTC--GTGTCAATTTATTGCCTCCTGTGTCGCTTATT----TAGTAC-------------------------------- 338
250 TGGTATTGGAGTGCTTTAGCCTTT--ATTACTTG-----TGTCAATTTCTTGCCTCCTGTGTCATTTCTT----T---------------------------------- 313
Consensus TGGTATTGGAGTGCTTTAGCCTTTTATTTACTTGTCATGTGTCAATTTATTGCCTCCTGTGTCATTTATT----TAGAACTCATTTTTCTTTTTCTTC+TACTTACAAACTA
pA
U-rich
GUGU
GUGU
