# Supplementary figures and images for: The Conserved Intronic Cleavage and Polyadenylation Site of CstF-77 Gene Imparts Control of 3′ End Processing Activity through Feedback Autoregulation and by U1 snRNP
Source: PLoS Genet. 2013 Jul 11;9(7):e1003613. doi: 10.1371/journal.pgen.1003613 (PMC3708835; doi:10.1371/journal.pgen.1003613)

Figure S2

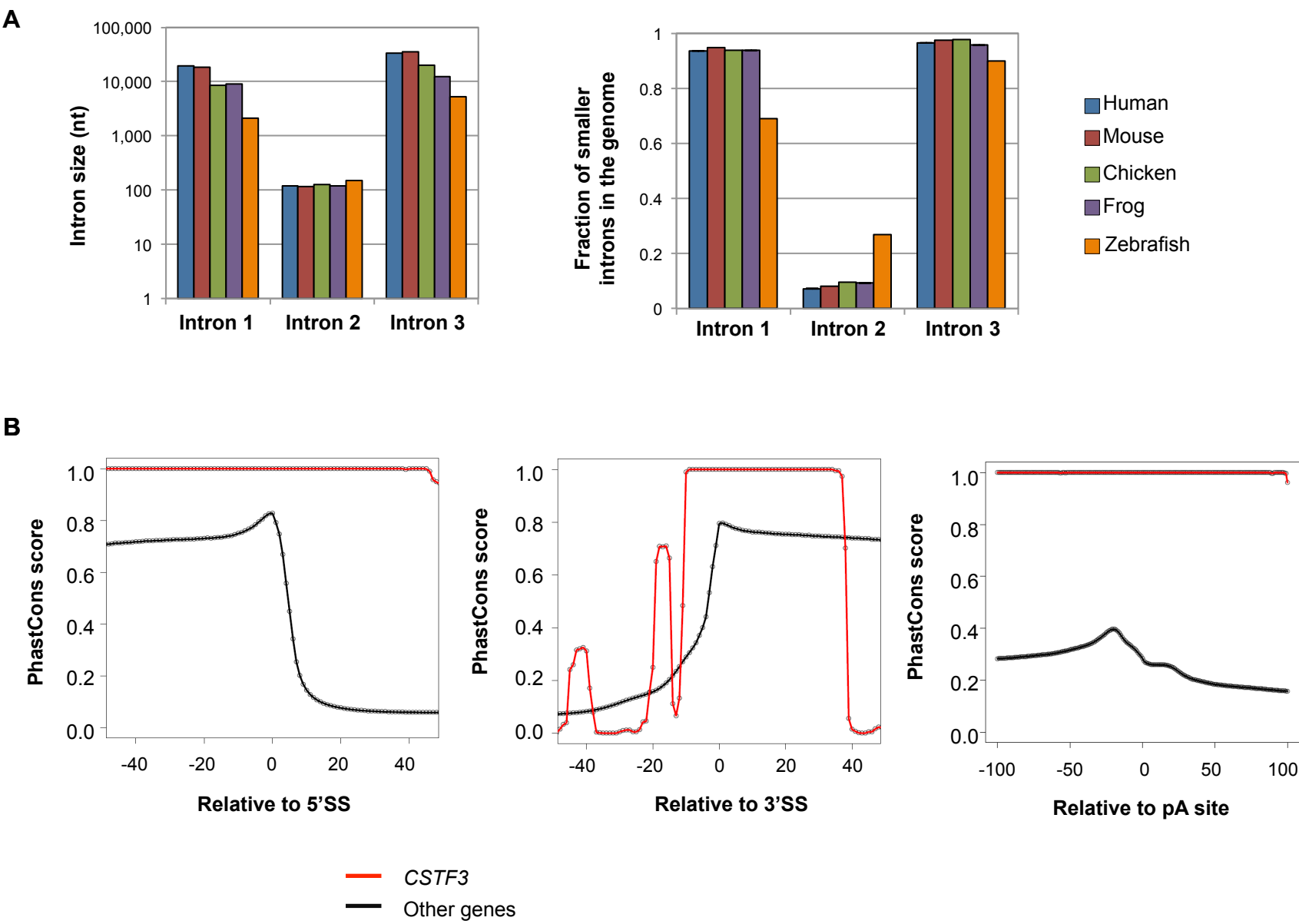

Supplement: Figure S2 — Conservation analysis of intron 3 of CSTF3. (A) Conservation of intron size for introns 1, 2 and 3 of CSTF3 across vertebrates. Left, intron size; right, fraction of introns smaller than the indicated intron in a specific species. (B) Conservation profiles around 5′SS (left), 3′SS (middle) and pA (right). PhastCons score distribution for each region was calculated based on 17 vertebrate species. Red lines represent CSTF3 and black lines represent all other RefSeq-supported introns in the human genome or all other pAs in the human genome reported in PolyA_DB 2. (PDF) [file pgen.1003613.s002.pdf]

Figure S3

A

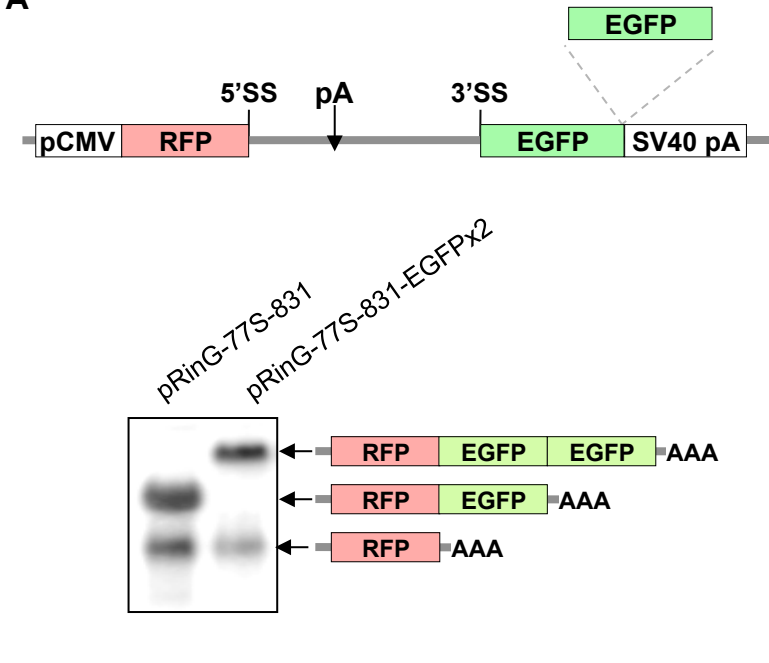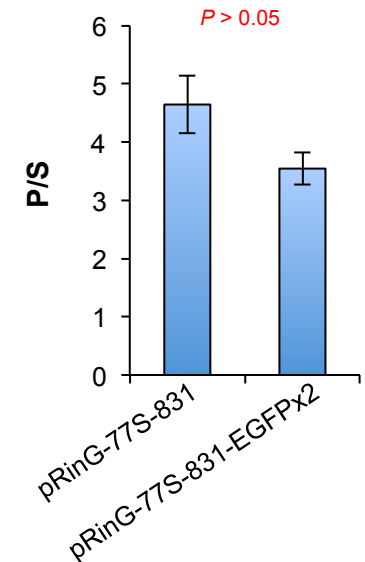

B

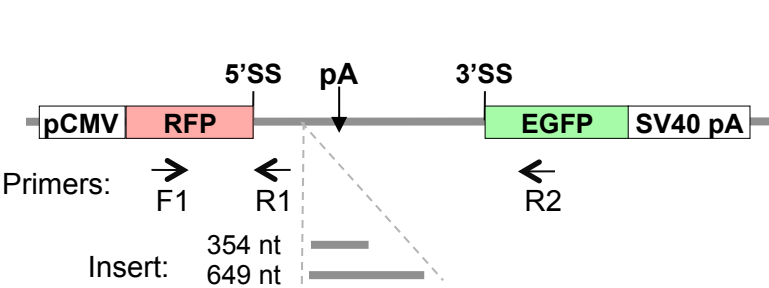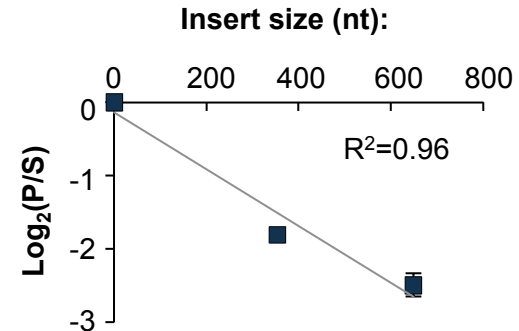

Supplement: Figure S3 — Analysis of the effect of the distance between the intronic pA and 3′-most pA or the 5′SS. (A) Left, Northern blot analysis of mRNA expressed from the pRinG-77S vectors containing an intronic insert of 831 nt and one or two EGFP sequences, as indicated in the graph; Right, quantification of the Northern blot data. (B) Left, two random sequences (354 nt and 649 nt) were inserted into the region between 5′SS and pA (indicated in the graph). Expression was analyzed by RT-qPCR using the indicated primer pairs. Right, quantification of isoform expression using RT-qPCR. (PDF) [file pgen.1003613.s003.pdf]

Figure S4

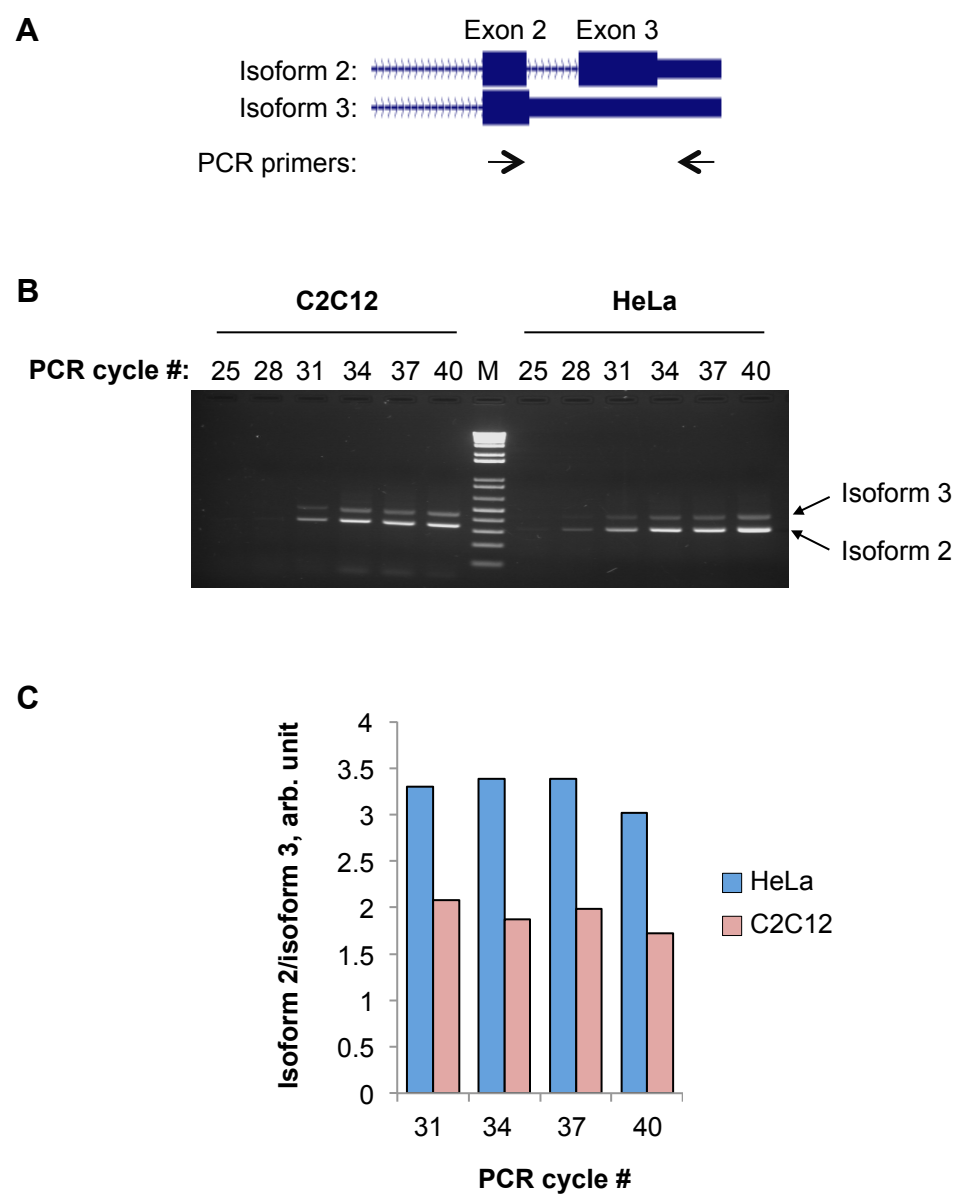

Supplement: Figure S4 — Analysis of isoforms 2 and 3 in HeLa and C2C12 cells. (A) Schematic showing primers used to examine isoforms 2 and 3. (B) RT-PCR products of isoforms 2 and 3 at different cycles of PCR. (C) Relative expression of isoforms 2 vs. 3 in HeLa and C2C12 cells. Quantification of expression was based on the RT-PCR products shown in (B). (PDF) [file pgen.1003613.s004.pdf]

Figure S5

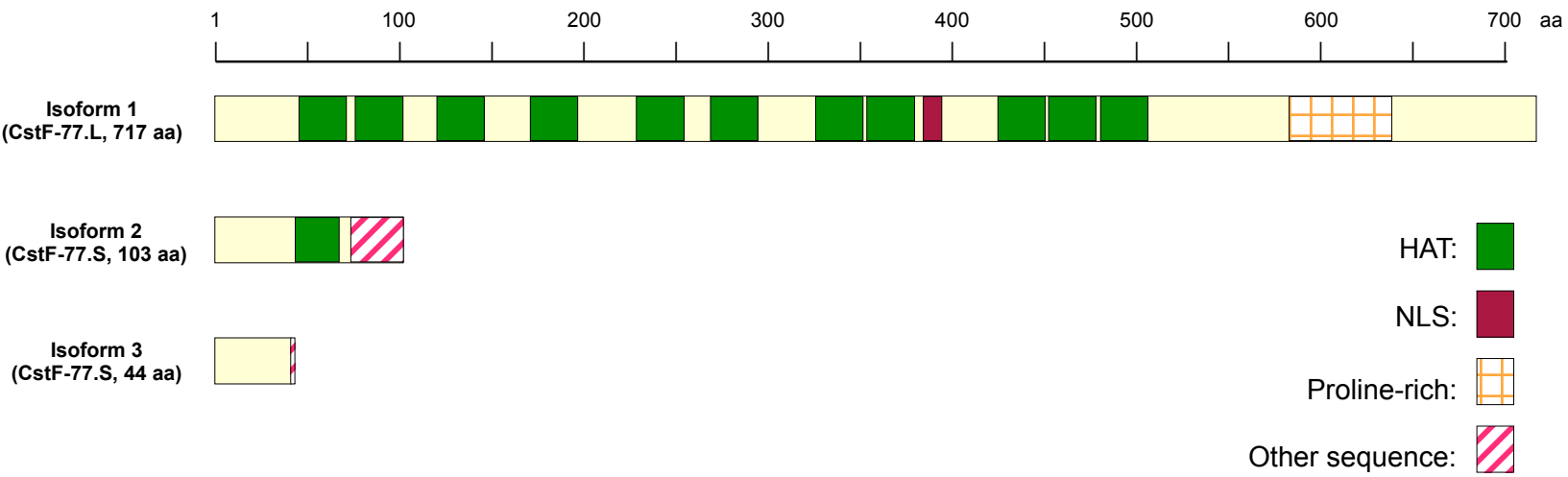

Supplement: Figure S5 — Protein sequences encoded in CstF-77 mRNA isoforms. Protein motifs and domains are indicated. (PDF) [file pgen.1003613.s005.pdf]

Figure S6

A

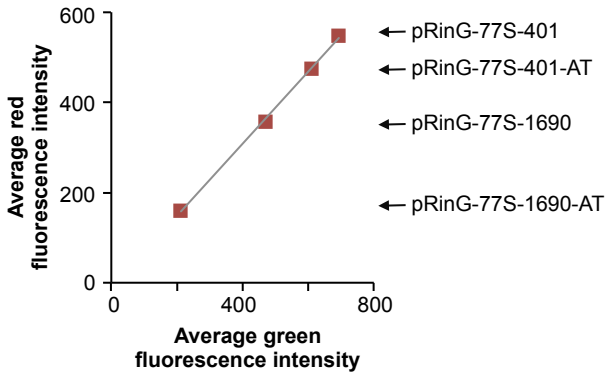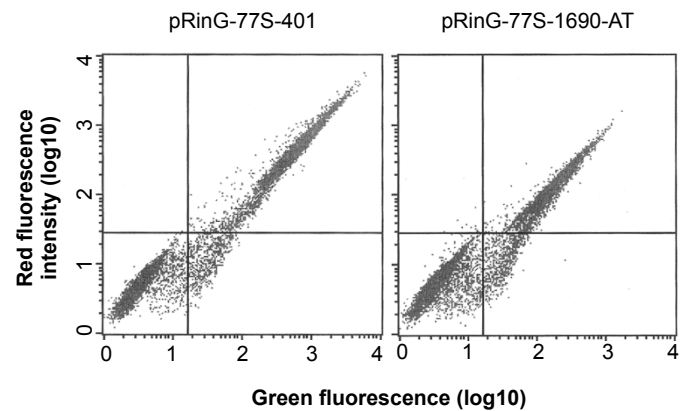

B

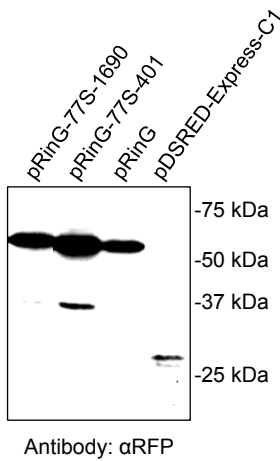

C

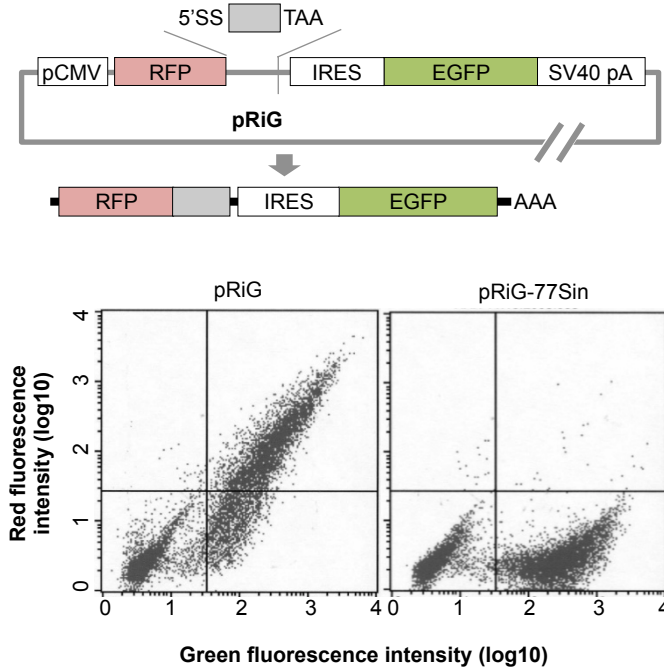

Supplement: Figure S6 — The coding sequence of intron 3 inhibits protein expression. (A) Average red fluorescent intensity vs. average green fluorescent intensity for HeLa cells transfected with different pRinG-77S constructs (Left). Right, examples of FACS analysis result for pRinG-77S-401 and pRinG-77S-1690-AT. (B) Immunoblot (IB) with HeLa cells expressing different constructs using antibody against RFP. The expected size of the protein product expressed from the intronic pA isoform of pRinG-77S is ∼30 kDa. Note: the protein of ∼37 kDa in the pRinG-77S-401 lane is likely to be a degradation product of the RFP-EGFP fusion protein (major band at ∼56 kDa). (C) Top, schematic of pRiG-77Sin, which contains the intronic sequence from 5′SS to stop codon inserted into the pRiG vector. Bottom, FACS analysis of HeLa cells transfected with pRiG and pRiG-77Sin. (PDF) [file pgen.1003613.s006.pdf]

Figure S7

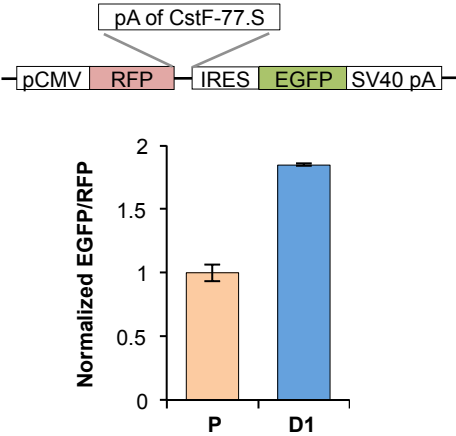

Supplement: Figure S7 — pA usage is inhibited in differentiating C2C12. Top, pRiG construct containing the intronic pA of CstF-77. Bottom, analysis of pA usage using pRiG. (PDF) [file pgen.1003613.s007.pdf]

Figure S8

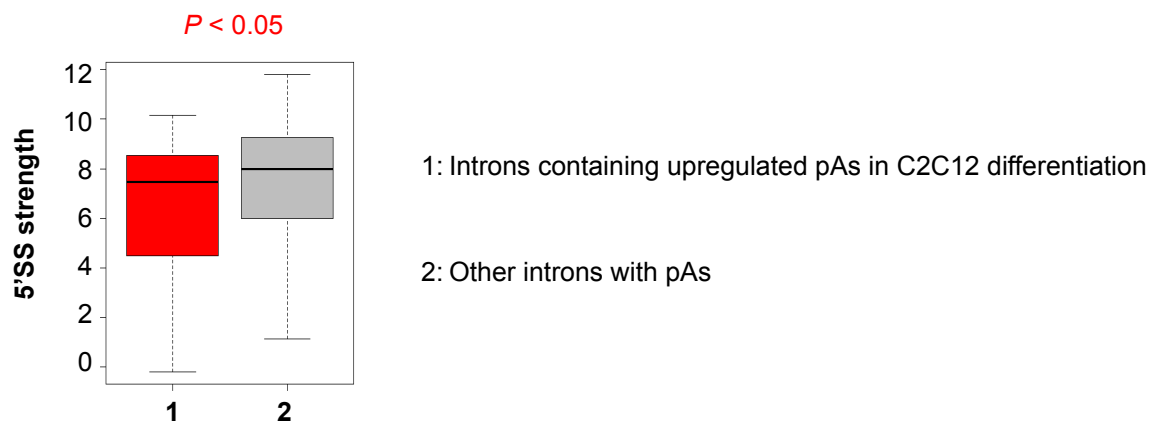

Supplement: Figure S8 — Introns containing pAs upregulated in C2C12 differentiation tend to have a weak 5′SS. Boxplot of 5′SS strength (MaxEnt score) for introns containing upregulated pAs (red) and other introns containing pAs (grey). (PDF) [file pgen.1003613.s008.pdf]

Figure S9

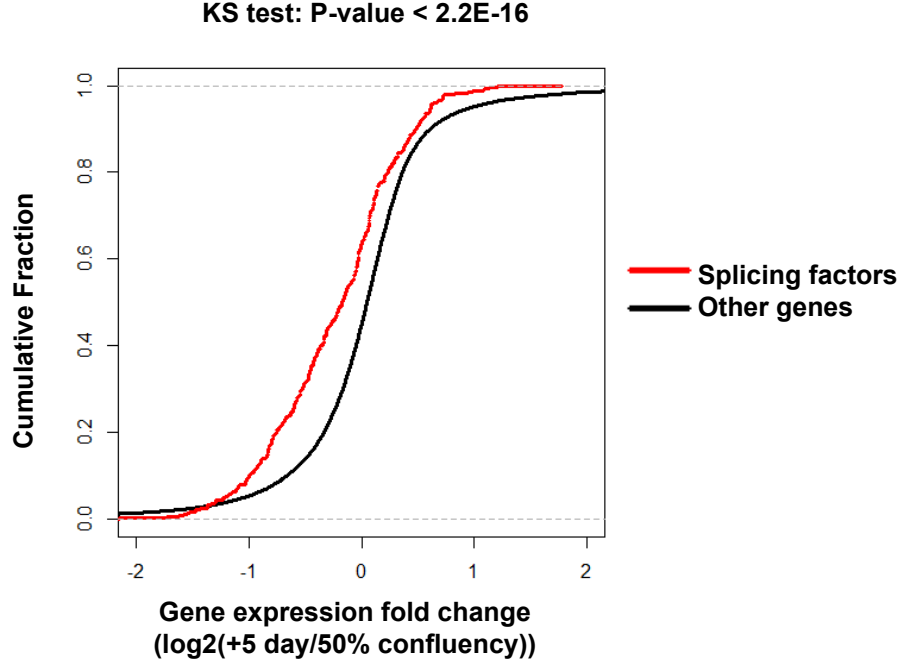

Supplement: Figure S9 — Splicing factors are generally downregulated in C2C12 differentiation. Cumulative fraction lines for gene expression changes of splicing factor genes (172 in total) and of other genes are shown. P-value based on the Kolmogorov-Smirnov (KS) test is shown, which compares the distributions of two gene sets. The data were derived from the microarray dataset GSE11415 in the GEO database. (PDF) [file pgen.1003613.s009.pdf]

Figure S10

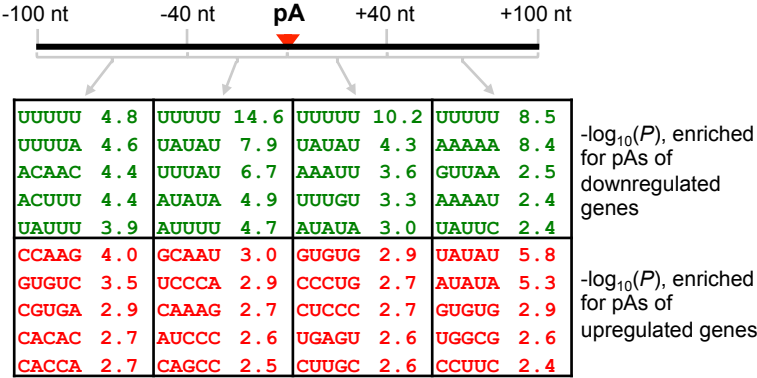

Supplement: Figure S10 — Pentamers enriched for pAs of downregulated (top) or upregulated genes (bottom). Only genes with a single pA were used for analysis. (PDF) [file pgen.1003613.s010.pdf]

Figure S11

A

All genes

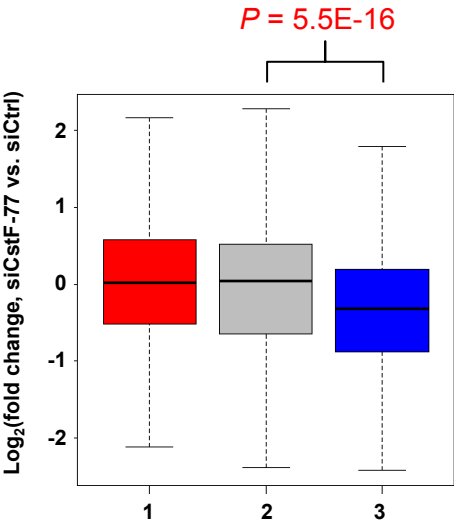

B

Cell cycle genes only

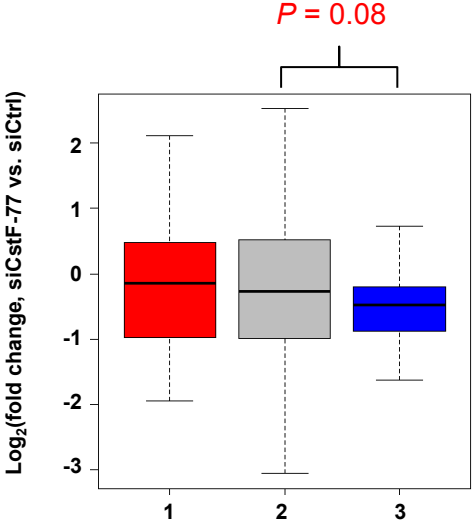

- 1: 3'UTR lengthened
- 2: No change
- 3: 3'UTR shortened

Supplement: Figure S11 — Gene expression changes vs. 3′UTR regulation after CstF-77 knockdown. (A) All genes. (B) Cell cycle genes. For each gene set, genes were divided into 3 groups based on 3′UTR regulation. P-values (Wilcoxon test) comparing two sets are shown. (PDF) [file pgen.1003613.s011.pdf]
