# Supplementary material for: The Conserved Intronic Cleavage and Polyadenylation Site of CstF-77 Gene Imparts Control of 3′ End Processing Activity through Feedback Autoregulation and by U1 snRNP
Source: PLoS Genet. 2013 Jul 11;9(7):e1003613. doi: 10.1371/journal.pgen.1003613 (PMC3708835; doi:10.1371/journal.pgen.1003613)
Supplement: Table S1 — Constructs used for reporter assays. (DOCX) [file pgen.1003613.s012.docx]

**Table S1. Constructs used for reporter assays**

| pRinG | A fragment containing the open reading frame (ORF) of EGFP was generated by PCR using pIRES2-EGFP (BD Biosciences) as template and primers 5’-cgatggatccATGGTGAGCAAGGGCGAG and 5’-ggccggatcccttgtacagctcgtccat. The fragment was cut by BamH I and then inserted into the pDsRED-Express-c1 vector (BD Biosciences) digested with the same enzymes. |
| --- | --- |
| pRinG-77S-5’SS | The genomic sequence including the 5’SS of exon 3 and pA of intron 3 in human *CSTF3* was amplified by PCR using genomic DNA from HeLa cells with primers 5’-CGATCTCGAGACATTGAAGCAGAGGTTACT and 5’-ggccgaattcATGTTTCATTTCACCAGAC. This sequence contains 14 nt of exon 3 and a 201 nt region downstream of the AUUAAA PAS. The PCR product was cut by Xho I and EcoR I and then inserted into the pRinG vector digested with same enzymes. |
| pRinG-77S-401  pRinG-77S-831  pRinG-77S-1690  pRinG-77S-2378 | The fragments containing different 3’ regions of intron 3 and a portion of exon 4 (27 nt) were generated from genomic DNA of HeLa cells using a common reverse primer 5’-GGCCGTCGACAACCTTGTCATAATTTTTAGC, and specific forward primers: 5’-cgatgaattcCTCAGATTGTTCTGGTAGC for pRinG-77S-401, 5’-cgatgaattcCCACTCTGTGATGAAAATAC for pRinG-77S-831, 5’-cgatgaattcAGGAGAAATACAACTGGAAC for pRinG-77S-1690 and 5’-cgatgaattcTGTAAGTTTTTGCCATTCTA for pRinG-77S-2378. The PCR products were cut by EcoR I and Sal I and then inserted into the pRinG-77S-5’SS digested with same enzymes. |
| pRinG-77S-401-AT  pRinG-77S-831-AT  pRinG-77S-1690-AT  pRinG-77S-2378-AT | The AUUAAA PAS was mutated to AAUAAA by the Phusion™ Site-Directed Mutagenesis Kit (NEB) according manufacturer’s protocol using primers 5’-tgaaactgttttattgttgttgcaact and 5’-atggtattggagtgctttagcctt. |
| pRinG-77S-401-noGU  pRinG-77S-831-noGU  pRinG-77S-1690-noGU  pRinG-77S-2378-noGU | For pA mutants without the downstream GU-rich elements, we first generated a PCR product using pRinG-77S-401 as template and primers 5’-CGATCTCGAGACATTGAAGCAGAGGTTACT and 5’-ggccgaattcACAAGTAAATAAAAGGCT, and used it to replace the sequence between 5’SS and pA in the template construct using Xho I and EcoR I. The inserted sequence lacks a 156 nt region downstream of the cleavage site. The intronic sequence containing 3’SS was replaced with corresponding sequences containing different fragments (831 nt, 1,690 nt, and 2,378 nt) by compatible restriction enzymes. |
| pRinG-77S-401-AT-noGU  pRinG-77S-831-AT-noGU  pRinG-77S-1690-AT-noGU  pRinG-77S-2378-AT-noGU | Same as pRinG-77S-401/831/1690/2378-noGU, except that pRinG-77S-401-AT was used as template for cloning. |
| pRinG-77S-401-AT-5’SSMT1  pRinG-77S-831-AT-5’SSMT1  pRinG-77S-1690-AT-5’SSMT1  pRinG-77S-2378-AT-5’SSMT1  pRinG-77S-401-AT-5’SSMT2  pRinG-77S-831-AT-5’SSMT2  pRinG-77S-1690-AT-5’SSMT2 | For constructs containing 5’SS mutations, we first introduced mutations to 5’SS by PCR using pRinG-77S-401-AT as template, a common reverse primer 5’-ggccgaattcATGTTTCATTTCACCAGAC, and two different forward primers, 5’- CGATCTCGAGACATTGAAGCAGAGGTAACTATTTTAT for mutant 1 (MT1) 1 and 5’CGATCTCGAGACATTGAAGCACAGGTAAGTATTTTAT for mutant 2 (MT2). PCR products were cut by Xho I and EcoR I and were used to replace corresponding sequences containing the wild type 5’SS in different vectors. The intronic sequence containing 3’SS was replaced with corresponding sequences containing different fragments (831 nt, 1,690 nt, and 2,378 nt) by compatible restriction enzymes. |
| pRinG-77S-401-TT-3’SSMT  pRinG-77S-831-TT-3’SSMT  pRinG-77S-1690-TT-3’SSMT | Constructs containing 3’SS mutations were introduced by PCR using a reverse primer 5’-GGCCGTCGACTTTAGCTTTAATCTCAAGAGAAACAG and different forward primers specific for the vectors with different intron sizes, which were used as templates. PCR products were cut by EcoR I and Sal I and then used to replace corresponding sequences containing the wild type 3’SS in different vectors. |
| pRinG-77S-831-EGFPX2 | To get double EGFP inserts in pRinG-77S-831, we generated a PCR fragment containing EGFP sequence using primers 5’-cgatgtcgacatggtgagcaagggcgag and 5’-ggccggatcccttgtacagctcgtccat. The PCR product was cut by Sal I and BamH I and inserted into the pRinG-77S-831 digested with the same enzymes. Another fragment containing the EGFP sequence was derived from pRinG and was inserted downstream of the first EGFP fragment using the BamH I site. |
| pRinG-77S-354-831  pRinG-77S-649-831 | To insert sequences between 5’SS and pA, we first created a Hind III site between 5’SS and pA as follows: a PCR product using pRinG-77S-831 as template and primers 5’-CGATCTCGAGACATTGAAGCAGAGGTTACT and 5’-ggccaagcttCTAAT TGGCTATGTTCCTAAC were cut by Xho I and Hind III and inserted into pRinG. We then inserted a PCR product that was amplified by 5’-cgataagcttGACAAGGAGGATTTAAATGTG and 5’-ggccgaattcATGTTTCATTTCACCAGAC and cut with Hind III and Sal I. We inserted sequences from intron 3 into the vector by PCR using HeLa genomic DNA as template and primers 5’-cgataagcttTGTAAGTTTTTG CCATTCTA and 5’-ggccaagcttttatattcctttctggttgg, followed by digestion with Hind III. Complete and partial digestions of this PCR product led to fragments containing 354 bp and 649 bp intron sequences, respectively. Fragments containing 3’SS and different intronic sequences were inserted into this vector using Eco I and Sal I sites. |
| pRiG-77Sin | We inserted into the pRiG vector a fragment containing ORF of RFP followed by a genomic sequence of *CSTF3* which starts from the 3’ end of exon 3 to the first in-frame stop codon in intron 3. The fragment was generated by PCR using pRinG-77S-401 as template and primers 5’- cgatgctagcATGGCCTCCTCCGAGGAC and 5’-GGCCGAATTCCTAATTGGCTATGTTCCTAAC. The fragment was cut by Nhe I and EcoR I and inserted into the pRiG cut with the same enzymes. |
