# Supplementary material for: The Conserved Intronic Cleavage and Polyadenylation Site of CstF-77 Gene Imparts Control of 3′ End Processing Activity through Feedback Autoregulation and by U1 snRNP
Source: PLoS Genet. 2013 Jul 11;9(7):e1003613. doi: 10.1371/journal.pgen.1003613 (PMC3708835; doi:10.1371/journal.pgen.1003613)
Supplement: Table S2 — siRNAs used in this study. All siRNAs are for mouse genes unless indicated otherwise. (DOCX) [file pgen.1003613.s013.docx]

**Table S2. siRNAs used in this study**

| **Name** | **Target sequence** |
| --- | --- |
| siCstF-77.L (human) | 5’-CCCUGAUAUUUGGUAUGAA |
| siCstF-77.S (human) | 5’-AAAUAGGUAUAAAGGAGUA |
| siCstF-77 | 5’-CGAGGAUUCAGACGAAGAU |
| siCstF-64 | 5’-GCACAGGUAGUGAUGAGAAUU |
| siCPSF-160 | 5’-CCGCAATCUUAUGGUGUAUAU |
| siCPSF-73 | 5’-CCAGCAAACCAGUGAAUUUAU |
| siCFI-25 | 5’-CGUCUCUAUGAGCACAGCUUA |
| siCFI-59 | 5’-GUCCUCAUCUCCUCUCUUA |
| siCFI-68 | 5’-CCUGUUGUAACUCCAUGCAAU |
| siU1-70K | 5’-CCCUCACAAUGAUCCCAAU |
| siSF3B1 | 5’-GUCACUUGGUGUUUACGGA |
| siU2AF65 | 5’-GUGAGUACGUGGACAUCAA |

All siRNAs are for mouse genes unless indicated otherwise.
