# Supplementary material for: The Conserved Intronic Cleavage and Polyadenylation Site of CstF-77 Gene Imparts Control of 3′ End Processing Activity through Feedback Autoregulation and by U1 snRNP
Source: PLoS Genet. 2013 Jul 11;9(7):e1003613. doi: 10.1371/journal.pgen.1003613 (PMC3708835; doi:10.1371/journal.pgen.1003613)
Supplement: Table S3 — Antibodies used in this study. (DOCX) [file pgen.1003613.s014.docx]

**Table S3. Antibodies used in this study**

| **Name** | **Source** |
| --- | --- |
| anti-RFP | Clontech Laboratories |
| anti-Omni tag | Santa Cruz Biotechnology |
| anti-CstF-77 | Santa Cruz Biotechnology |
| anti-CstF-64 | Gift from Dr. Clinton C. MacDonald, Texas Tech University |
| anti-CPSF-160 | Abcam |
| anti-CPSF-73 | Bethyl Laboratories |
| anti-CFI-25 | Santa Cruz Biotechnology |
| anti-CFI-59 | Bethyl Laboratories |
| anti-CFI-68 | Santa Cruz Biotechnology |
| anti-U1-70K | Santa Cruz Biotechnology |
| anti-SF3B1 | Bethyl Laboratories |
| anti-U2AF65 | Santa Cruz Biotechnology |
