# Supplementary material for: The Conserved Intronic Cleavage and Polyadenylation Site of CstF-77 Gene Imparts Control of 3′ End Processing Activity through Feedback Autoregulation and by U1 snRNP
Source: PLoS Genet. 2013 Jul 11;9(7):e1003613. doi: 10.1371/journal.pgen.1003613 (PMC3708835; doi:10.1371/journal.pgen.1003613)
Supplement: Table S4 — RT-qPCR primers used in this study. (DOCX) [file pgen.1003613.s015.docx]

**Table S4. RT-qPCR primers used in this study**

| **Transcript/Gene Name** | **Sequence** |
| --- | --- |
| Isoform S | 5’-CCCGGCTACTACTACGTGGA; 5′-CTATCACTACAGTGAATGCTGCAA |
| Isoform P | 5’-CCCGGCTACTACTACGTGGA; 5′-GGACACGCTGAACTTGTTGG |
| Human CstF-77.S | 5'-GAGGCCATGTCAGGAGAC; 5'-CTATCACTACAGTGAATGCTGCAA |
| Mouse CstF-77.S | 5'-GAGGCCATGTCAGGAGAC; 5'-GCTGTAATTGCCATCAGATGCTA |
| Human and mouse CstF-77.L | 5'-GAGGCCATGTCAGGAGAC; 5'-CATAAATCAATGTGCAAAACC |
| *Ccnb1* | 5’-ACCAGAGGTGGAACTTGCTGAG; 5’-CCATCATCTGCGTCTACGTCAC |
| *Cdca3* | 5’-AGACGAAAAGCAAACAGCAAGG; 5’-GATTTTCCTTGTCGTGGTCCTG |
| *Cdk4* | 5’-AGTTCGTGAGGTGGCCTTGTTA; 5’-GGAGGTGCTTTGTCCAGGTATG |
| *Mcm6* | 5’-AGAACCAAATGACGGTGAAGGA; 5’-CTTGGATCACCAACAATGCAAA |
| *Tipin* | 5’-GCACTGGGCACATAGGCTATTC; 5’-GGGCCATTAGCTTCTCCAACTT |
| *Myh3* | 5’-TCAGGAAAGCCCAGCATGAGC; 5’-CTGCACCAGGAGGTCTTGCTC |
| *MyoG* | 5’-CCAGCGAGGGAATTTAGCTGACTC; 5’-CCCTGCCTGTTCCCGGTATCATCA |
| *Tpm2* | 5’-GAGAAAACCATTGATGATCTGGAAG; 5’-CAGTGGGGACTCAGAGGGAAG |
